# Free Energy of Membrane Pore Formation and Stability from Molecular Dynamics Simulations

**DOI:** 10.1021/acs.jcim.4c01960

**Published:** 2025-01-10

**Authors:** Timothée Rivel, Denys Biriukov, Ivo Kabelka, Robert Vácha

**Affiliations:** † Central European Institute of Technology, Masaryk University, Kamenice 5, CZ-62500 Brno, Czech Republic; ‡ National Centre for Biomolecular Research, Faculty of Science, Masaryk University, Kamenice 5, CZ-62500 Brno, Czech Republic; § Department of Condensed Matter Physics, Faculty of Science, Masaryk University, Kotlářská 267/2, CZ-61137 Brno, Czech Republic

## Abstract

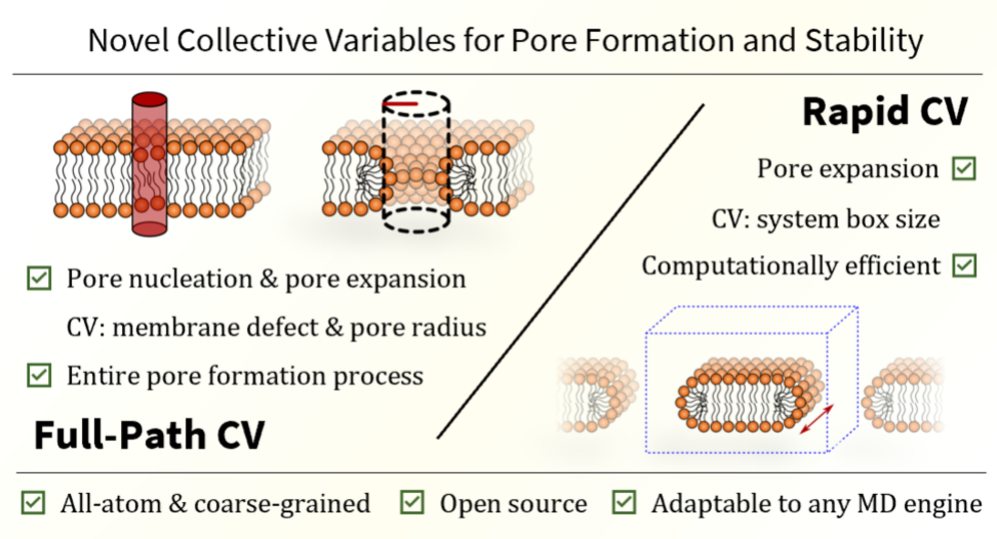

Understanding the molecular mechanisms of pore formation is crucial for elucidating fundamental biological processes and developing therapeutic strategies, such as the design of drug delivery systems and antimicrobial agents. Although experimental methods can provide valuable information, they often lack the temporal and spatial resolution necessary to fully capture the dynamic stages of pore formation. In this study, we present two novel collective variables (CVs) designed to characterize membrane pore behavior, particularly its energetics, through molecular dynamics (MD) simulations. The first CV—termed Full-Path—effectively tracks both the nucleation and expansion phases of pore formation. The second CV—called Rapid—is tailored to accurately assess pore expansion in the limit of large pores, providing quick and reliable method for evaluating membrane line tension under various conditions. Our results clearly demonstrate that the line tension predictions from both our CVs are in excellent agreement. Moreover, these predictions align qualitatively with available experimental data. Specifically, they reflect higher line tension of 1-palmitoyl-2-oleoyl-*sn*-glycero-3-phosphocholine (POPC) membranes containing 1-palmitoyl-2-oleoyl-*sn*-glycero-3-phospho-l-serine (POPS) lipids compared to pure POPC, the decrease in line tension of POPC vesicles as the 1-palmitoyl-2-oleoyl-*sn*-glycero-3-phosphoglycerol (POPG) content increases, and higher line tension when ionic concentration is increased. Notably, these experimental trends are accurately captured only by the all-atom CHARMM36 and prosECCo75 force fields. In contrast, the all-atom Slipids force field, along with the coarse-grained Martini 2.2, Martini 2.2 polarizable, and Martini 3 models, show varying degrees of agreement with experiments. Our developed CVs can be adapted to various MD simulation engines for studying pore formation, with potential implications in membrane biophysics. They are also applicable to simulations involving external agents, offering an efficient alternative to existing methodologies.

## Introduction

Pore formation in cellular membranes is a crucial phenomenon to understand cellular defense mechanisms and to design novel therapeutic strategies. For instance, antimicrobial peptides can be engineered to induce pore formation in lipid membranes, compromising the cellular barrier function.^1^ The resulting uncontrolled exchange of matter has severe consequences for intracellular processes, often leading to the death of bacteria, viruses, or other target cells.^2^ Furthermore, investigating pore formation provides valuable insights into the fundamental principles of cellular biology, such as the transport mechanisms of water-soluble molecules across lipid membranes, and can facilitate the controlled delivery of larger biomolecules, such as through electroporation.^3^

Unfortunately, capturing transient structures of membrane pores experimentally is extremely challenging. Some information about pore size and atomic-scale features can be determined from neutron scattering,^4−6^ solid-state NMR,^7−9^ atomic force microscopy,^10,11^ or conductivity measurements.^12^ In some cases, even the entire three-dimensional structure of the pore can be successfully resolved by means of X-ray crystallography.^13^ However, the static snapshots usually obtained from these methods are insufficient to fully describe the molecular mechanisms of pore formation and its subsequent stability.

At the same time, a lot of structural information about membrane pores can be obtained using computer modeling and particularly molecular dynamics (MD) simulations.^14^ Due to the slow lipid diffusion and long time scales involved in pore-formation processes, it is beneficial to perform MD simulations applying enhanced sampling methods. These methods allow us to determine the free energy landscapes of pore formation,^15,16^ providing critical insights into the evolution of pore structures. However, defining a unique collective variable (CV) that accurately describes the whole pore formation process is not straightforward. Previous approaches have generally suffered from hysteresis, imposed constraints on pore topology, convergence issues, and simulation artifacts.^17^ Moreover, the pore formation process could involve two distinct conformational regimes—nucleation and expansion—which are difficult to describe accurately using conventional CVs due to the inherent complexity of capturing both stages in a unified manner.

In one of the more successful approaches, Hub and Awasthi proposed a CV based on tracking the distribution of water and phosphorus polar atoms to facilitate the formation of a continuous water channel.^18^ This CV was designed to follow pore nucleation process, and, as such, it was not suitable for monitoring the subsequent stages of pore expansion. To address this limitation, a refined approach was developed, combining the original CV for pore nucleation with an additional CV tailored specifically for pore expansion.^19^ In the regime of pore expansion, the free energy is primarily attributed to a linear term *G*(*r*) = 2π*r*γ, where *r* is the pore radius and γ (also noted as σ in some works) is the line tension associated with the pore/membrane edge. Nevertheless, further refinement of existing CVs is still necessary to improve their ability to accurately capture pore formation, especially given the complexity of the nucleation process. For example, Bubnis and Grubmüller^16^ demonstrated that it is difficult to pinpoint the specific contributions of different atomic groups, such as water, lipid head groups, or lipid tails, in the formation of membrane defects during pore nucleation.

In this work, we propose two novel CVs to describe pore behavior in lipid membranes, implemented through the freely available PLUMED library^20^ to enhance compatibility with various MD engines. Our first CV (referred to hereafter as Full-Path) focuses on tracking the distribution of hydrophobic lipid tails rather than water molecules and lipid head groups. Our second CV—hereafter referred to as Rapid—is designed to rapidly estimate the line tension by modeling an “infinite pore” and using the box size as the biased parameter. While the latter CV is not suitable for probing pore nucleation, it is an excellent choice for providing quick and computationally cheap estimates of the stability of larger pores, even at the atomistic level. We demonstrate the accuracy of the pore structures identified by these CVs and calculate the associated changes in free energy by applying the umbrella sampling (US) method. Both all-atom and coarse-grained models were tested, and the resulting free energy profiles are compared. Importantly, we revealed no hysteresis during pore opening and closing. The results obtained using both CVs were successfully compared to each other and recent experimental data, demonstrating that MD simulations can effectively describe pore formation, evolution, and stability.

## Methods

### Full-Path Method: Pore Formation and Expansion

#### Definition of the Collective Variable

**Figure 1:**
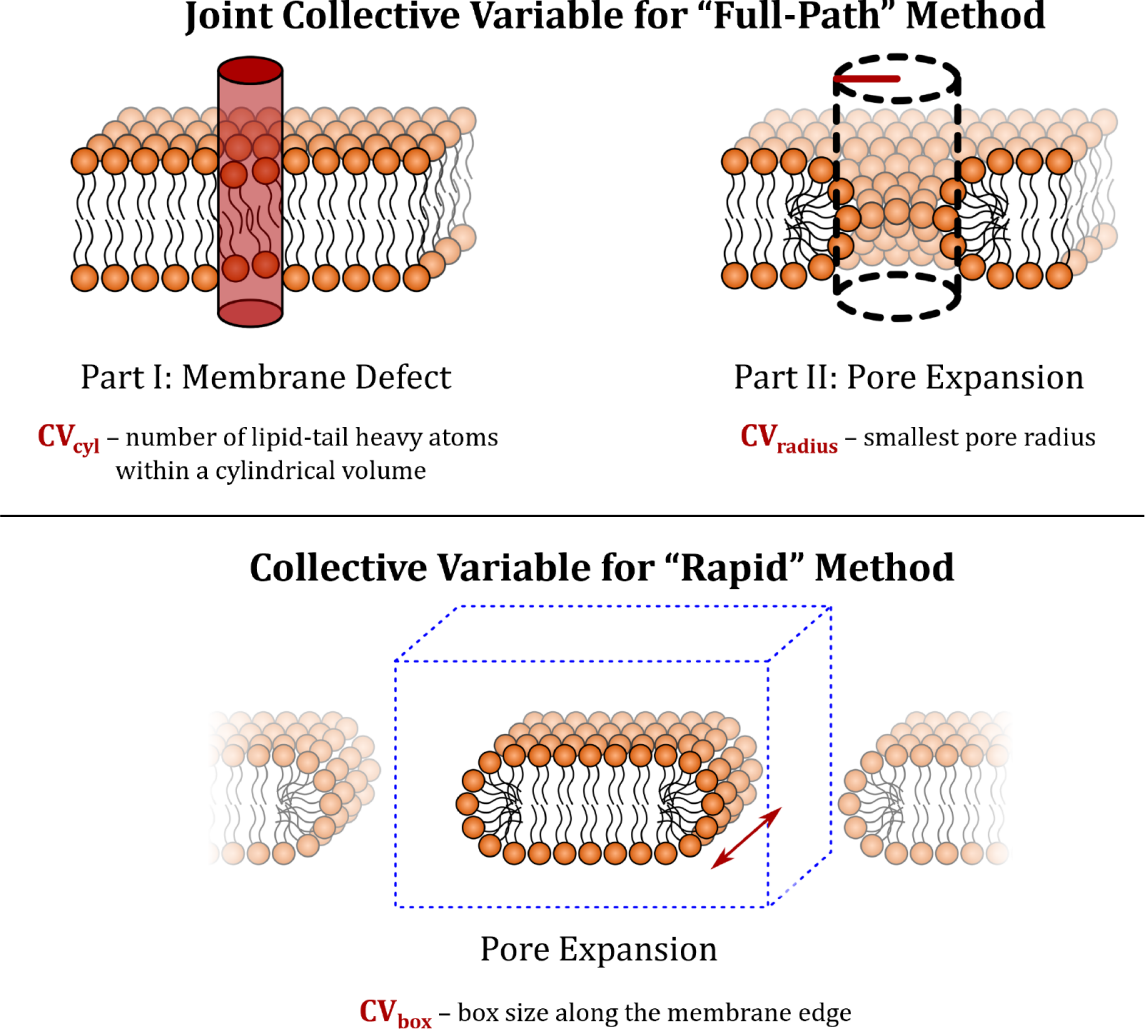
Schematic representation of the collective variables introduced in this work. The upper panel presents the joint Full-Path CV, which consists of two parts describing pore nucleation and pore expansion, respectively. Pore nucleation is characterized by the formation of a defect modeled by a change in the density of aliphatic carbons within a local cylinder. Pore expansion is characterized by the increase in the minimum distance between the center of the pore and surrounding aliphatic carbons. The lower panel present the Rapid CV that uses a lipid stripe to model an “infinite” toroidal pore. The change in the membrane edge length (pore rim) is controlled by adjusting the size of the simulation box along the axis parallel to the pore.

Our Full-Path CV is implemented through the PLUMED library^20^ and has been tested in combination with the GROMACS simulation engine.^21^ This Full-Path CV comprises two components: (1) the formation of a membrane defect (CV_cyl_) and (2) the expansion of the pore (CV_radius_), see Figure 1. The CV is implemented as the weighted sum of its individual parts:
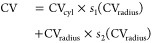
1where *s*_1_ and *s*_2_ are complementary switching functions:
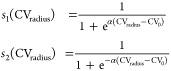
2The function *s*_1_ and *s*_2_ intersect at CV_0_, while the parameter α defines the rate at which these functions transition from 0 to 1, see Figure S2 in the Supporting Information.

The first component of the CV—membrane defect, CV_cyl_—is characterized by the number of lipid-tail heavy atoms within a cylindrical volume of radius *R*_cyl_. The optimal value of *R*_cyl_, along with the optimization of CV_0_ and α, is further discussed in the Results and Discussion section. Specifically, the PLUMED actions INCYLINDER and DENSITY are used to calculate the number of atoms inside a given virtual cylinder. This cylinder is centered in the system and aligned along the *z* axis, spanning the entire simulation box. The calculated number of atoms *d* is then converted to represent the size of the membrane defect:
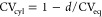
3where CV_eq_ is the number of lipid-tail heavy atoms inside the defined cylinder in an equilibrated intact bilayer. To ensure that the functional form of dimensionless CV_cyl_ is continuously differentiable for all real values, a smoothly decaying RATIONAL switching function is used.

The second part of the CV—pore expansion, CV_radius_—is defined as the smallest pore radius *r*_min_. This radius is calculated between the center of the pore and the closest lipid-tail heavy atom in the *xy* plane using the PLUMED action XYDISTANCES. The minimum value is estimated using a converging sum over all distances, ensuring that the CV remains continuously differentiable. The final expression of CV_radius_ is then rendered dimensionless, CV_radius_ = *r*_min_/*r*_unit_, by dividing the value of the minimal distance by the radius of a pore with *r*_unit_ = 1 nm.

Consequently, our joint CV can be interpreted as follows: values below the switching threshold, CV_0_, represent the extent of the membrane hydrophilic defect, whereas values above this threshold correspond to the radius of the hydrophilic pore.

#### Free Energy Simulations

Each modeled lipid bilayer was constructed using CHARMM-GUI online utility.^22−24^ All-atom bilayers comprised 200 lipids in total (100 per leaflet), while coarse-grained bilayers contained 600 lipids. To ensure proper equilibration, each bilayer underwent a series of preparatory steps suggested by CHARMM-GUI developers. This included an initial energy minimization, six equilibration simulations with gradually released position restraints, and preproduction run of 300 ns for all-atom systems and 1000 ns for coarse-grained ones.

Simulations were performed using GROMACS software,^21^ versions 2020.3, 2021.4, and 2022.3, compiled with the PLUMED libraries versions 2.7 and 2.8.^20^ Various force fields were tested, namely all-atom CHARMM36,^25,26^ and coarse-grained Martini 2.2,^27^ Martini 3,^28^ and Martini 2.2 polarizable.^29^ CHARMM-specific TIP(S)3P water model^30,31^ was used in CHARMM36 simulations, a polarizable Martini coarse-grained water model^29^ in Martini 2.2p simulations, and corresponding coarse-grained Martini water models for simulations with Martini 2.2^27^ and Martini 3 force fields.^28^ A minimum of 50 water molecules or 20 Martini water beads per lipid were added in the all-atom and coarse-grained systems, respectively. A physiological concentration of ∼0.15 M NaCl salt was added and simulated with the default force-field-specific ion parameters. The parameters for the simulations with the different force fields are described in Tables S2 and S3, including the treatment of interactions^32−35^ and used thermostats^36−38^ and barostats.^39,40^ Lipid and solvent atoms were always assigned to two separate temperature-coupling groups maintained at 310 K. Semi-isotropic coupling scheme was employed for pressure control at 1 bar. The time step was set to 2 and 20 fs for all-atom and coarse-grained simulations, respectively.

Free energy calculations associated with the Full-Path CV were performed using umbrella sampling method with a force constant κ = 5000 kJ mol^–1^. In all-atom simulations, evenly spaced windows—with the CV values ranging from −0.100 to 2.175 with an interval of 0.035—were generated from a 100 ns steered MD simulation. We used the weighted histogram analysis method (WHAM) to calculate free energy profiles from the umbrella sampling simulations, following a script by D. Bauer,^41^ which was adapted from Grossfield’s implementation.^42^ Free energy profiles were derived from the last 50 ns of the 200 ns production runs for each window as the initial 150 ns were necessary for the convergence of the free energy profiles.^19^ For Martini simulations,^27^ the membrane pore was opened over 1 μs. The windows were generated in the same way as for all-atom simulations, but a microsecond-long production run was used for each window, with the last 250 ns used for the free energy calculations. To assess their convergence, we calculated the free energy profiles using 20 ns parts of the trajectory for all-atom simulations and 250 ns parts of the trajectory for coarse-grained simulations (see Figure S4), showing only marginal differences. To test the CV hysteresis, the protocol was reversed, as shown in Figure S3. The quadratic and the linear parts of the free energy profiles were fitted by means of a linear least squared regression on , where , *k* is later referred to as the quadratic coefficient, and *c* is the *y*-intercept. We fitted the quadratic region for values where CV ≤ 0.5, while the line tension γ was fitted for values where CV ≥ 1.2. The errors for both *k* and γ were calculated as half the difference between the coefficients calculated from the first and second halves of the production simulation time.

#### Spontaneous Pore Closure Simulations

While spontaneous pore opening in pure membranes is very unlikely to occur during the microsecond time scale of simulations, pore closure is relatively easy to set up and follow. Assuming that pore opening and closure follow the same pathway, we performed, for the design of the Full-Path CV, simulations of spontaneous pore closure. Up to four lipid bilayers of phosphatidylcholine (PC) with different lengths and saturation levels of their fatty acids were used, including dimyristic acids (14:0, DM), dipalmitic acids (16:0, DP), dioleic acids (18:1, DO), and palmitic and oleic acids (16:0–18:1, PO). The temperature was kept at 310 K except for DPPC where it was set at 323 K to avoid gel phase. All simulations were performed with membranes composed of 256 lipids solvated in at least 90 water molecules per lipid. Lipids were distributed equally among both leaflets. Sodium and chloride ions were added at isotonic concentration of 150 mM. To create a plain lipid bilayer, we used an in-house script which placed the required amount of lipids on a grid. To prepare reference structure of a membrane pore, lipids inside a circular area were removed from the structure of the plain lipid bilayer. System was then solvated and appropriate amount of ions (to keep 150 mM NaCl solution) was added. For subsequent production MD simulations, semi-isotropic pressure coupling was used. After energy minimization, a first simulation in the canonical ensemble with 0.1 fs time step was followed by four simulations in *NpT* with an increasing time step. During this series of equilibration simulations, position restraints were applied to the lipids, pore area, and water molecules. Restraints on the lipids were used to control the relaxation process of the lipids and ensure the formation of a flat lipid bilayer. Restraints on the pore were used to keep the pore opened. Restraints on water molecules were used only during the first simulation of this series, performed in the canonical ensemble, to allow the membrane to relax before water could enter the pore region. The first equilibration step had a time step of 0.1 fs, while the next simulations, performed in the isothermal–isobaric ensemble, used a time step of 0.5, 1, and 2 fs, respectively. A preproduction run of 300 ns with restraints only on the pore radius was then used. Initially, the pore had diameter of about 4.5 nm. For each system, a number of replicas (see Table S1) was generated using the last 100 ns of the simulations used to equilibrate the systems with a pore.

Simulations were performed using GROMACS software,^21^ versions 2016.2, compiled with the PLUMED library version 2.7.^20^ To test the influence of the force field, different all-atom force fields were tested, namely Berger,^43^ CHARMM36,^25,26^ Lipid14,^44^ and Slipids.^45−48^ The simulation parameters used with the different force fields can be found in Tables S2 and S3. Overall, the main simulation details were consistent with those used in production runs for free energy calculations. Spontaneous pore closure simulations were analyzed using in-house scripts based on PLUMED library^20^ and MDAnalysis.^49^

**Figure 2:**
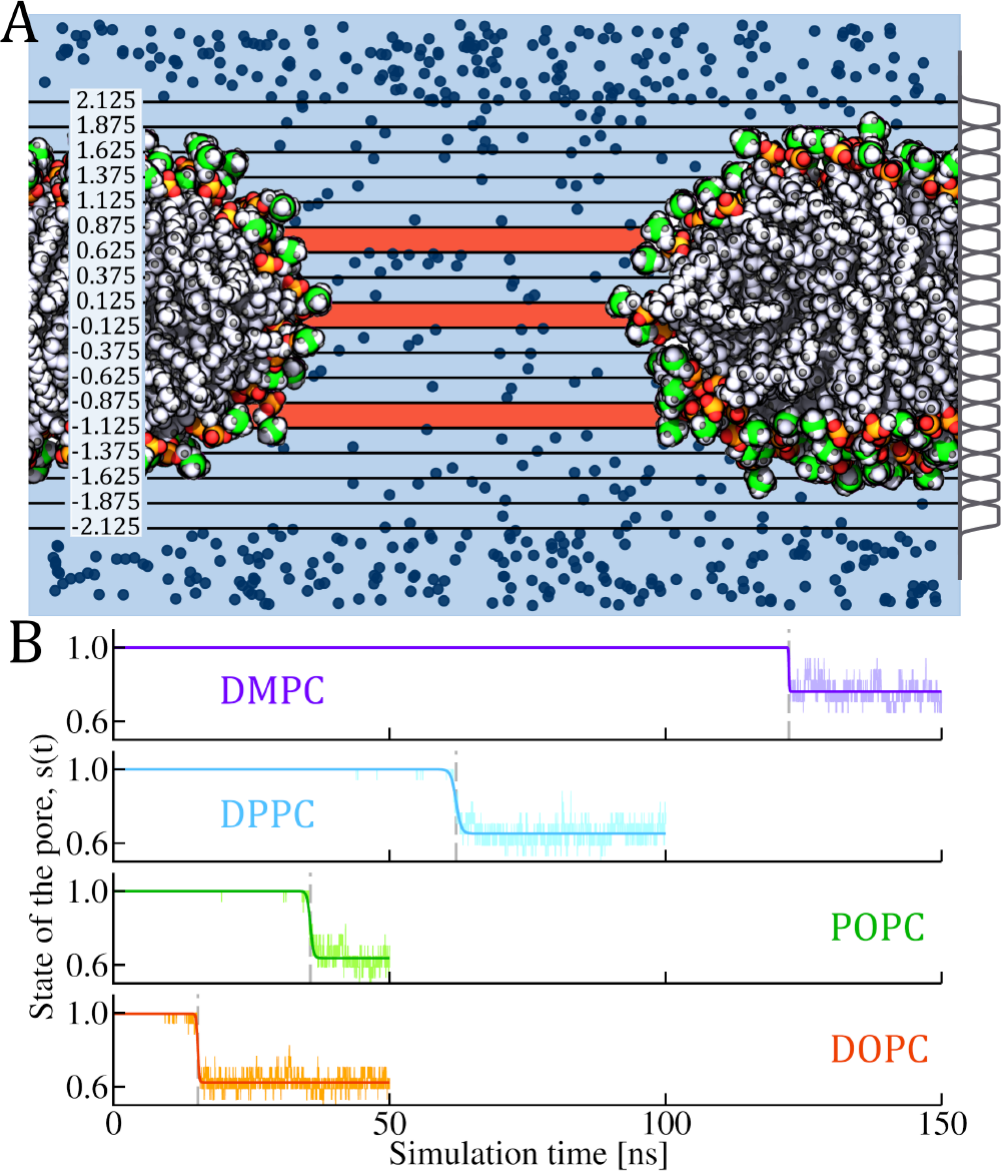
(A) Schematic illustration of pore opening and the evaluation of its state. The system is split in slices parallel to the membrane plane. Black lines depict slice boundaries with values denoting the distance from the membrane center. In each slice, the number of water molecules is evaluated by applying the weight function shown in gray on the right of each slice. Blue slices contain at least one water molecule, while vermilion slices were determined as without water. The number of slices containing water molecules indicates the state of the pore opening, *s*(*t*), see Methods for details. Background VMD snapshot captures opened membrane pore. The dark blue markers represent positions of water molecules. (B) Time evolution of the custom-defined state of the pore, *s*(*t*), for four model membranes simulated with CHARMM36 force field, for which spontaneous pore closure events were investigated. For clarity, only one replica per membrane is shown. The state of the pore tracks the transition between a membrane with and without a pore. This transition occurs when water molecules no longer form a continuous thread across the membrane. For each membrane, the measured observable from the simulations is shown in lighter color. In darker color, the fit of *s*(*t*) is displayed, following the equation , where *A*_2_ is the pore lifetime and *A*_0_ and *A*_1_ are the optimized parameters. Light gray vertical dashed lines indicate the calculated pore lifetime, representing the time at which the pore closure event occurs.

To analyze spontaneous pore closure events, we defined pore lifetime by fitting a timeline of pore opening (see Table 1). Pore opening was captured by the pore state, *s*(*t*), implemented in PLUMED library (see Figure 2A). To calculate the pore state, we first split the membrane in slices of 0.25 nm along the normal to the membrane, i.e., *z* axis. The slices are comprised within the interval [−2.125, 2.125] nm and centered around the center of geometry of the lipid bilayer. This interval, which slightly exceeds the membrane thickness, allows us to account for natural membrane undulations. Each slice is defined by selecting the atoms whose positions along the *z* axis fall within the boundaries of the slice. To smooth the atomic positions, we use the so-called GAUSSIAN function defined in PLUMED, which gives greater weight to atoms nearer the center of the slice. We then applied a Heaviside step function, , to each slice *s*_*i*_(*t*) from the *N*_S_ slices, assigning a value of 0 if a slice contains fewer than one molecule, and 1 otherwise. Finally, we computed the average value to follow the pore state over simulation time . Figure 2B shows the time evolution of this observable, that we refer to as *s*(*t*), for one replica of each of the four model membranes investigated using the CHARMM36 force field. We fitted this observable for all the simulated systems with a hyperbolic tangent function , where *A*_2_ is the pore lifetime, corresponding to the inflection point of the function, and *A*_0_ and *A*_1_ are the optimized parameters.

**Table 1:** Pore Lifetime (in ns) in Various Lipid Bilayers Modeled Using Different Force Fields^a^

force field	DMPC	DPPC	POPC	DOPC	ref
CHARMM36	122 (N/A)	94 (40)	34 (5)	15 (1)	this work
Lipid14			63 (16)	24 (5)	this work
Berger	134 (16)		156 (71)	131 (43)	this work
Slipids	110 (24)	32 (5)	27 (4)	18 (2)	this work
Berger	123	6			(59)
Slipids			30	20	(60)

a The average values from multiple replicas are provided (where available), and the standard error is indicated in parentheses (if applicable). N/A stands for non-applicable (due to lack of measurements). The values reported in the previous studies are added for comparison.

### Rapid Method: Quick Estimation of Line Tension

#### Definition of the Collective Variable

The Rapid method uses a lipid stripe to model a pore with “infinite” size, as illustrated in Figure 1. The stripe was created by extending a simulation box of equilibrated lipid bilayer along one axis of membrane plane. The lipids at the membrane edge in contact with aqueous solution quickly reorient to reduce the contact between hydrophobic tails and surrounding aqueous environment. In this configuration, the lipid stripe features two distinct membrane edges creating rims of infinite pore formed via periodic boundary conditions (PBC). The free energy associated with the length of the rim is characterized by the line tension γ.

To evaluate the line tension, we conducted umbrella sampling simulations using the box size along the rim as the CV. The range of gradually changing box sizes was modeled, with the box size restrained in each simulation using the PLUMED action RESTRAINT. The collected free energy profile was then fitted with a linear model, with the slope corresponding to the line tension γ as γ = *m*/(2 × *N*_A_), where *m* is the slope, *N*_A_ is the Avogadro constant, and the division by two accounts for the presence of two pore rims. The error was calculated as half the difference between the line tension values calculated from the first and second halves of the production simulation time.

#### Simulation Methods, Models, and Parameters

Each lipid composition was initially constructed as a lipid bilayer using CHARMM-GUI.^22−24^ The bilayers were then equilibrated following the CHARMM-GUI-recommended simulation protocol, which included energy minimization, six equilibration simulations with gradually released position restraints, and a short production run. Then, the bilayers were placed in a larger box, where one of the membrane lateral dimensions is extended. Following equilibration simulations were performed until the formation and stabilization of the lipid stripe, with a detailed procedure described in the SI. Once all position restraints on lipid atoms were removed, flat-bottom restraints with a force constant *k*_fb_ = 1000 kJ·mol^–1^·nm^–2^ were introduced to prevent the stripe from rotating and self-interact with itself through PBC. The reference for the flat-bottom restraint was calculated as the membrane normal coordinate of the stripe’s center of mass (in this case, *x* coordinate). The flat-bottom potential was acting on lipid phosphorus atoms when their *x* distance from the center of mass was larger than 2.5 nm, which accounts for the leaflet thickness and several hydration layers. Note that, in all lipid-stripe simulations, the box dimension along the *x* axis, parallel to the membrane normal, was set to 8.5 nm. This approach minimized the size of the modeled system without impacting the properties of the bilayer. Additionally, in all systems tested, the distance between the two rims was at least 2 nm, guaranteeing that the pore is sufficiently large to prevent any previously reported artifacts due to PBC.^19,50^

To generate multiple initial configurations/windows with varied rim sizes along the pore, the membrane was compressed. Similar to the Full-Path method, free energy calculations for the Rapid method were performed using umbrella sampling simulations with a force constant of 5000 kJ·mol^–1^·nm^–2^. A total of 21 evenly spaced windows were used to cover box sizes ranging from 6 to 6.6 nm. In principle, generating the linear dependence requires only two umbrella sampling simulations. However, to mitigate potential errors due to insufficient sampling, we employed a larger number of windows, as the computational costs of these simulations are modest. In the SI, we demonstrate that using every other window yields very similar line tension (Figure S5); moreover, it is likely that even fewer windows could be needed when simulating a smaller range of box sizes or when using every third or fourth window with a weaker force constant. The gmx wham utility^51^ was used to derive free energy profiles. The free energy profiles were calculated using the final 100 ns of the 150 ns production runs for each window in both the all-atom and Martini simulations. Simulations were performed using GROMACS software,^21^ version 2022.3, compiled with the PLUMED library version 2.8.^20^

Using the Rapid method, we tested a variety of lipid compositions, see Table S5 for full summary. We cross-compared line tension predictions for these compositions using various models: all-atom CHARMM36,^25,26^ prosECCo75 (a CHARMM-based force field incorporating electronic polarization via charge scaling^52^), and Slipids,^45−48^ as well as coarse-grained Martini 2.2,^27^ Martini 2.2 polarizable^29^ (which is, in case of lipids, Martini 2.2 immersed in polarizable Martini water^29^), and Martini 3.^28^ Each lipid bilayer consisted of 200 lipids, solvated in either 20,000 water molecules or 5000 Martini water beads. In the case of standard Martini 2.2, 10% of the water beads were replaced with antifreeze particles.

CHARMM-specific TIP(S)3P water model^30,31^ was used in CHARMM36 and prosECCo75 simulations, original TIP3P water model in Slipids simulations, a polarizable Martini coarse-grained water model^29^ in Martini 2.2p simulations, and corresponding coarse-grained Martini water models for simulations with Martini 2.2^27^ and Martini 3 force fields.^28^ We examined two ionic conditions: one with a 0.15 m concentration of NaCl (note the use of molality for unambiguous, nonvolume-dependent force field comparison, unlike the molarity used in the Full-Path simulations) and the other with no ions (apart from the sodium counterions necessary to neutralize the net charge of anionic lipids). For further comparison with the available experimental data on giant unilamellar vesicles (GUVs),^53^ we performed a few additional simulations with 0.15 m concentration of CaCl_2_ instead, keeping Na^+^ as counterions for anionic lipids. Default ion parameters were used in simulations with CHARMM36, Martini 2, Martini 3, and Martini 2.2p, default AMBER ff99 force field ion parameters^54,55^ for simulations with Slipids, and recommended ion parameters for simulations with prosECCo75 (“NA_s”, “Cl_2s”, and “Ca_s”, see https://gitlab.com/sparkly/prosecco/prosECCo75). In total, using the Rapid method, we conducted simulations totaling ≈150 μs at the all-atom level and ≈155 μs at the coarse-grained level. A summary about all tested systems is provided in the SI, while simulation parameters were consistent with those used in the Full-Path simulations and summarized in Tables S2 and S3.

## Results and Discussion

### Pore Formation Characterized by the Full-Path Collective Variable

#### Development of the Full-Path Collective Variable

The most straightforward CVs describe pore formation by expanding the pore size, e.g., by increasing its radius or area. Such CVs have been effectively applied in coarse-grained simulations.^56−58^ However, recent studies have highlighted the significance of membrane defect formation as a crucial step in the pore-formation process.^18^ Therefore, we constructed our CV as a combination of two parts: (1) defect formation and (2) pore expansion, similarly to a recent work by Hub.^19^ In order to do that, we first identified which system-bound reaction coordinates play a driving role.

Spontaneous pore formation in pure lipid membranes is highly unlikely to occur within the microsecond time scales typically used in simulations.^59^ In contrast, pore closure is relatively easy to set up and follow. Assuming that the mechanisms of pore opening and closing share a common pathway, we can study the dynamics of pore closure to gain valuable insights into the process of pore formation. These insights can, in turn, inform the development of the CV. For spontaneous pore closure simulations, we therefore used four model membranes, namely DMPC, DPPC, POPC, and DOPC, to account for the effect of the degree of saturation and fatty acid length on the process of pore formation. We simulated these four membranes using a variety of all-atoms and united-atoms force fields, specifically Berger,^43^ CHARMM36,^25,26^ Lipid14,^44^ and Slipids.^45−48^

**Figure 3:**
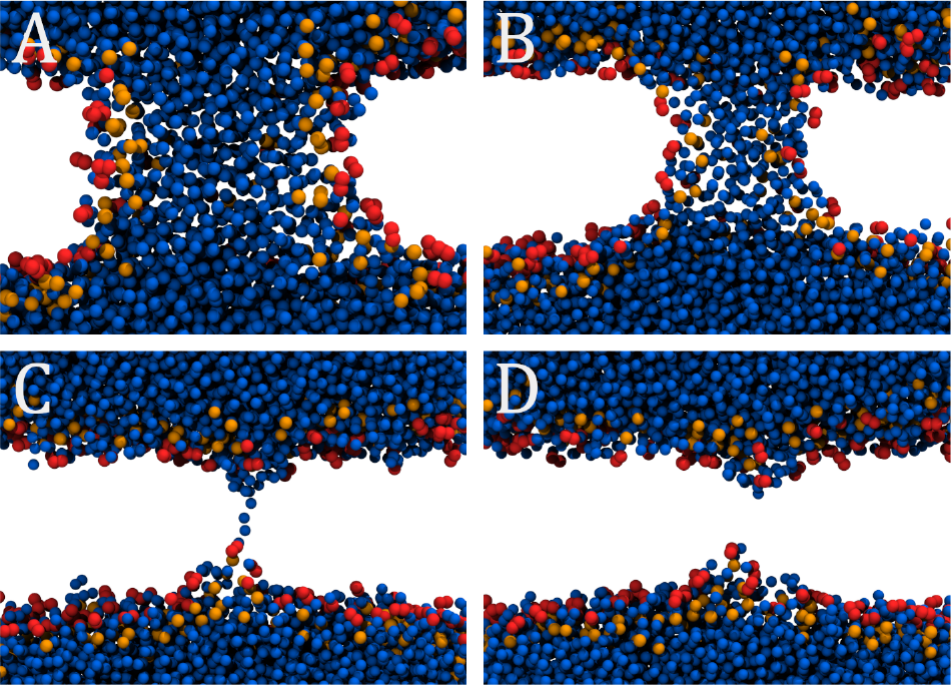
Snapshots depicting spontaneous closing of a pre-equilibrated membrane pore. Cross-section through the pore is displayed for: (A) initial structure of the equilibrated pore. (B) Snapshot of the pore during closure, where the overall structure is maintained but the radius is shrinking. (C) Last frame of the spontaneous pore closure that shows a continuous water thread defining the pore. (D) Lipid bilayer after pore closure, indicating an early stage of pore (de)nucleation with localized membrane thinning. Water beads are shown in blue, while lipid head groups and carbonyl groups are shown in orange and red, respectively. Lipid tails are not shown for clarity.

Figure 3 illustrates several snapshots of the pore-closing process, capturing its key events. Upon visual inspection, the process can be divided into two distinct stages. In the first stage, the size/radius of the continuous water channel gradually decreases, but the channel remains preserved, while lipid head groups and carbonyl groups participate in stabilizing the pore. In the second stage, the water channel is broken, and only membrane defects (with water molecules penetrating deeply into the bilayer) persist. Importantly, lipid carbonyl groups remain in place of the former water channel and interact with the solvent. As the process continues, the water defects diminish, and the lipid head groups return to their unperturbed equilibrium structure.

Table 1 shows the measured pore lifetimes τ for our systems (see Methods for details) and compares them with similar estimates from the previous studies. The previously reported lifetimes were estimated differently than in our work, yet they are consistent with our findings. Additionally, due to the high variability between replicates, we primarily focus on the observed trends. Specifically, the general trend is τ_DMPC_ > τ_DPPC_ > τ_POPC_ > τ_DOPC_, suggesting that pore stability is negatively correlated with both fatty acid length and saturation degree. This correlation also agrees with the available experimental data.^61^ The exception is DMPC simulated with the Berger force field; however, this could be attributed to numerous issues identified for this force field.^62,63^ Therefore, we can conclude that pores are generally more stable in membranes made of longer and more saturated lipids.

Interestingly, we observed that the average density in the distal carbon atom(s) of lipid tails positively correlates with the estimated pore lifetimes, Figure S6 in the SI. This observation prompted us to consider whether a depletion in the local density of lipid tails might be a suitable descriptor of membrane defects. This description represents an alternative to the previous CV focused on polar heavy atoms, namely oxygen from water and lipid phosphate groups.^19^ Notably, a recent work by Bubnis and Grubmüller^16^ suggested that pore nucleation in lipid membranes may involve contributions from lipid head groups, tails, and water molecules. However, formulation of such multicomponent CVs is very challenging. Thus, in this manuscript, we focused on the role of lipid tails (within both all-atom and coarse-grained models), following our observations from the spontaneous pore closure simulations.

To ensure that our CV accurately tracks the transition from a membrane defect to a membrane pore—as assessed using *s*(*t*)—we calculated the CV value at the pore lifetime for each replica (see Figure S7 in the SI). Our results show that the transition consistently occurs at CV_cyl_ values below 0.5 across all replicas. The distribution of CV_cyl_ is tightly centered around a single peak, indicating that this variable reliably captures the defect-to-pore transition. For comparison, we also plotted an alternative CV,^18^ which values at the defect-to-pore junction are significantly broader.

Next, we optimized the parameters CV_0_ and α for the Full-Path CV. Based on our findings, we defined a restricted optimization range for CV_0_, which should be greater than 0.5 to allow pore formation, but still below 1, which is the upper limit of CV_cyl_. Through steered MD simulations across all model membranes, we systematically varied these parameters to ensure the consistent behavior across the tested systems. We found that when CV_radius_ < 0.85, systems often remained near the maximum CV_cyl_ value without transitioning to the pore expansion regime. Ultimately, the optimal parameters were α = 20 and CV_0_ ∈ [0.90;0.95], as they provided the most linear response to the moving biasing potential across all tested force fields and membrane models.

To assess the sensitivity of our results to the cylinder parameters, we conducted several simulations using different cylinder radii *R*_cyl_ (0.5, 0.75, 1.5, and 3 nm). Figure S8 shows snapshots where a continuous water channel forms. From these snapshots, it is evident that a cylinder radius of 0.5 nm is insufficient, as it fails to fully form a continuous water channel. In contrast, cylinders with radii of 0.75 and 1.5 nm facilitate the formation of water channels that are comparable in size and characteristics to those observed in simulations of spontaneous pore closure. When the cylinder radius is too large (3 nm), the displacement of lipid tail atoms leads to significant membrane thinning before the formation of the water channel (see Figure S8). Therefore, using cylinder radii ≤ 0.5 or ≥ 3 nm would likely result in poor sampling and/or significant hysteresis. Importantly, the set of selected parameters (*R*_cyl_ = 1.2, α = 20, and CV_0_ = 0.95) revealed no hysteresis, see Figure S3 in the SI, indicating that these parameters could be used also for other phospholipids, although further testing may be necessary for very different lipid types, such as glycolipids. Overall, we demonstrate that we can successfully implement an alternative version of a CV describing pore formation in lipid membranes, which exhibits no hysteresis and relies on atom groups other than lipid head groups or water to form membrane defects.

#### Energetics of Pore Formation with the Full-Path Method

**Figure 4:**
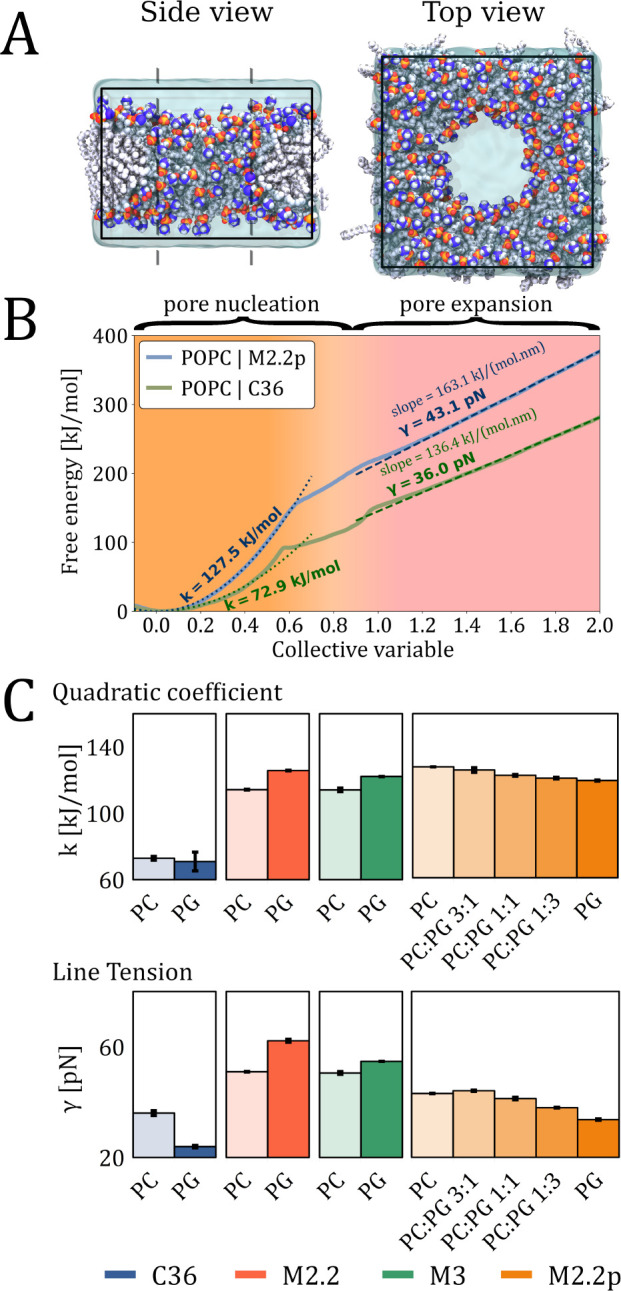
(A) Representative snapshots of the side and top views of membrane pore in MD simulations using the Full-Path method. Carbon, phosphorus, nitrogen, oxygen, and hydrogen atoms are shown as light gray, orange, indigo, red, and gray spheres, respectively. Water is shown as a semi-transparent cyan volume. The side view shows a cross-section through the middle of the pore. (B) Free energy profiles obtained from US simulations with the Full-Path CV. The solid lines represent the energy profiles, while the dashed lines correspond to the fits of the pore nucleation and pore expansion, respectively. The fitted quadratic coefficient *k* and line tension γ are given next to the free energy profiles. (C) Comparison of the quadratic coefficients *k* (top) and line tension γ (bottom) for POPC, POPG, and POPC:POPG bilayers calculated with different force fields.

To test our Full-Path method, we applied the developed CV to a set of simple yet biologically relevant model lipid membranes simulated using various force fields. A comprehensive summary of these systems is provided in Table S4 in the SI. Figure 4A shows representative snapshots of a POPC membrane with a well-defined pore simulated with the CHARMM36 force field. For all the systems, we computed the free energy profile of pore formation in order to encompass both pore nucleation and pore expansion regimes. Our free energy profiles (see Figure 4B for an example) demonstrate that the pore formation process can be indeed divided into two distinct regimes. The first regime follows a quadratic growth law, corresponding to the pore nucleation, while the second regime shows a linear trend indicative of pore expansion. An intermediate region displays more complex shapes in the free energy profiles due to contributions of both CV_cyl_ and CV_radius_. We also observe that the free energy profiles for pore formation are consistently lower when using all-atom force fields compared to the Martini family of coarse-grained force fields. This behavior has been reported in the literature before^64^ and may be attributed to the higher line tensions and bending moduli of membranes in coarse-grained systems.

To further test our CV, we evaluated the correlations between the observed variations in the quadratic coefficient *k* in the pore nucleation regime and line tension γ in the pore expansion regime. We selected lipid mixtures consisting of anionic (POPG or POPS) and zwitterionic (POPC or POPE) lipids as a representative set of systems. Understanding the behavior of these lipid mixtures is important in various biological contexts, such as bacterial cells (rich in PE and PG lipids), malaria-infected red blood cells (enriched in PS), or eukaryotic cells in general (that predominantly contain PC lipids). Our results summarized in Figures 4C and S9 reveal that differences in the quadratic coefficient *k* and the line tension γ are related. Specifically, we observe that a decrease in the quadratic coefficient is consistently correlated with a decrease in line tension (for the lipid compositions studied), and vice versa, see Figure S10 in the SI.

Furthermore, we observed that these correlations are dependent on the choice of the force field. For the CHARMM36 and Martini 2.2p force fields, both *k* and γ are lower for POPG bilayers compared to POPC bilayers. This trend is also evident in mixtures of these lipids, where the gradual addition of POPG lipids to POPC bilayers results in a decrease in both *k* and γ. In contrast, Martini 2.2 and Martini 3 show an opposite trend of POPG lipids having higher values of *k* and γ than POPC. Previous studies,^50^ including experimental measurements of line tension,^53,65,66^ demonstrated that the energy cost of pore formation in lipid membranes containing PG lipids is lower compared to PC lipids. Therefore, Martini 2.2 and Martini 3 force fields do not seem to correctly capture the lipid differences in the energetics of pore formation.

### Efficient Predictions of Line Tension by the Rapid Method

**Figure 5:**
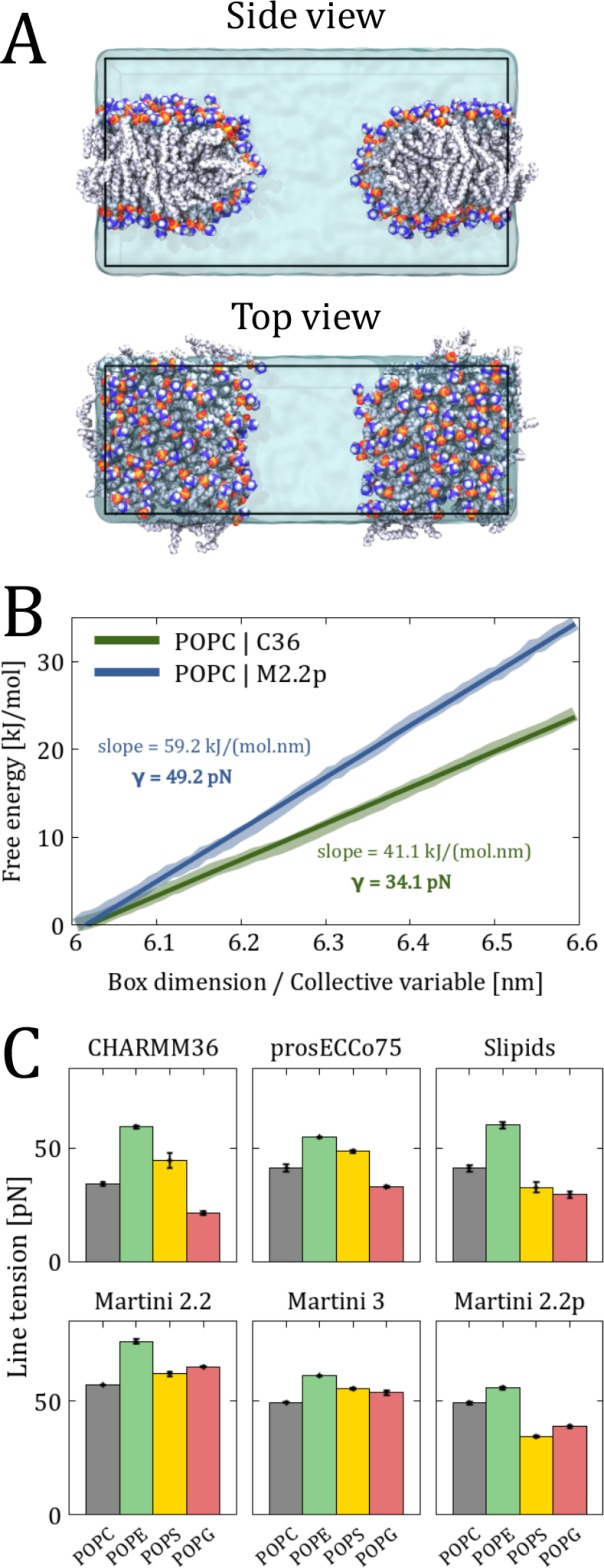
(A) Representative snapshots of the side and top views of a system setup for simulations with the Rapid method. Carbon, phosphorus, nitrogen, oxygen, and hydrogen atoms are shown as light gray, orange, indigo, red, and gray spheres, respectively. Water is shown as a semi-transparent cyan volume. (B) Free energy profiles obtained from US simulations with the Rapid CV. The thicker and lighter lines represent the energy profiles, while thinner and darker lines correspond to the linear fits. The slopes and calculated line tensions γ are shown next to the free energy profiles. The error—calculated using bootstrap analysis with 200 bootstrap samples as implemented in the gmx wham utility^51^—is thinner than the free energy profiles. (C) Comparison of the calculated line tensions for POPC, POPE, POPS, and POPG lipid stripes using different force field models.

**Figure 6:**
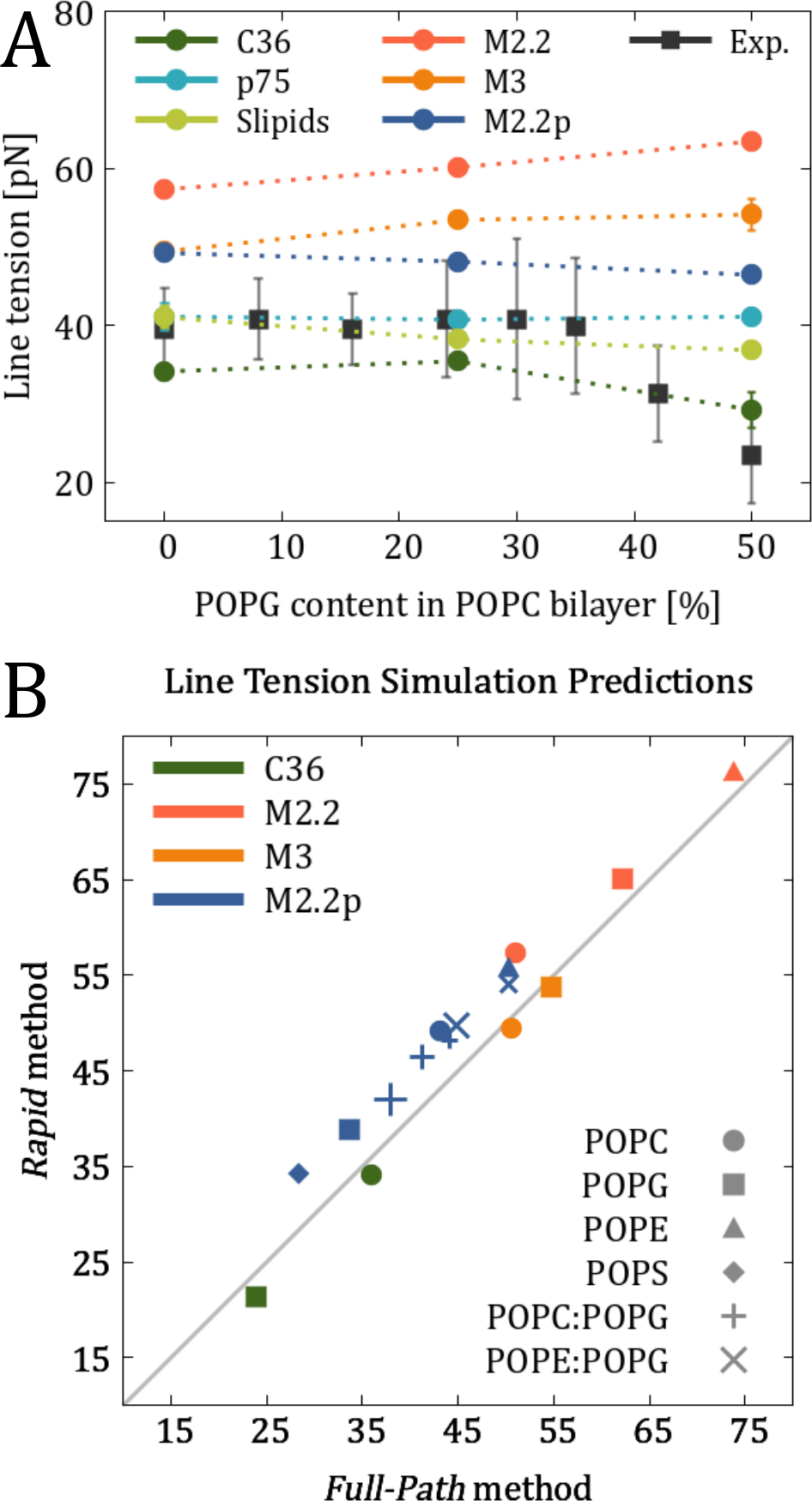
(A) Comparison of the calculated line tensions for POPC bilayers with various amounts of POPG lipids against reference experimental data from ref (53). The estimated error for MD data is not visible when the error bars are smaller than the size of the data symbols. (B) Comparison of line tension predictions from the Full-Path and Rapid methods. For mixtures, larger marker sizes indicate a higher proportion of POPG lipids in the lipid bilayer/stripe. The error bars are smaller than the markers and thus not shown for clarity.

**Figure 7:**
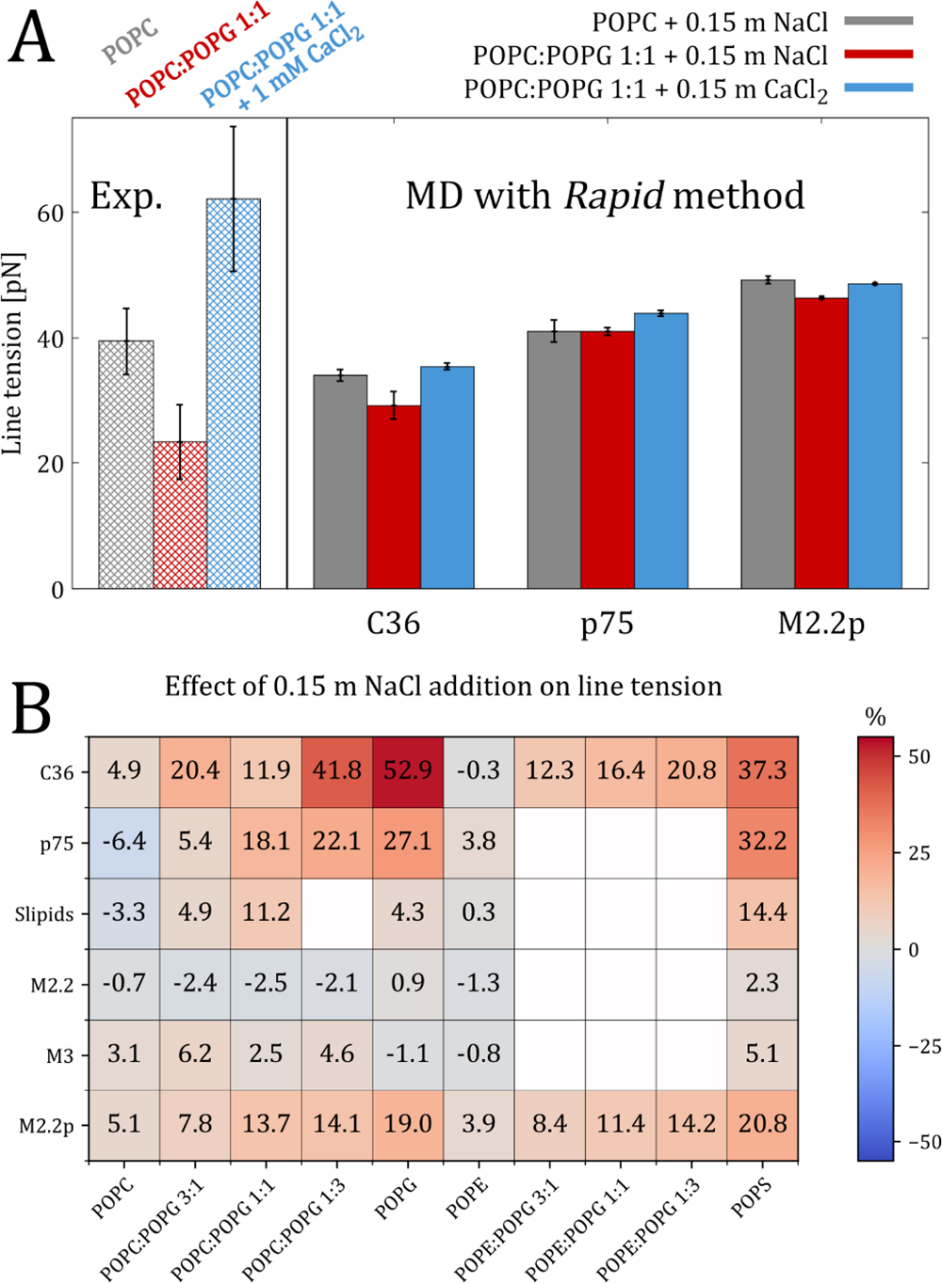
(A) Line tension changes upon incorporating 50 mol % POPG lipids into a POPC bilayer, and the effect of the subsequent addition of CaCl_2_. Experimental data from ref (53) are shown for comparison. (B) Effect of 0.15 m NaCl on the line tension of various lipid compositions employing different force fields.

We systematically investigated the pore formation trends between different lipids and force fields using the Rapid method. This method provides computational efficiency in evaluating membrane line tensions, which can be directly compared to experimental data. Figure 5A shows representative snapshots of an all-atom POPC stripe designed to simulate an infinite pore. Corresponding free energy profiles—collected as a function of the box size along the membrane rim—are shown in Figure 5B. These profiles exhibit a striking linearity, consistent with previous studies^19,50^ indicating that a linear regime is achieved for sufficiently large pores. Therefore, we converted the linear slope of these profiles into the line tension γ. All line tensions collected using the Rapid method are summarized in Figures 5C, 6, and 7.

First, we calculated the line tension of POPC, POPE, POPS, and POPG bilayers using the Rapid method with six different force fields. The results, presented in Figure 5C, clearly demonstrate notable differences in the performance of these force fields. All-atom force fields CHARMM36 and prosECCo75 consistently show a trend in which line tension decreases in the order of POPE > POPS > POPC > POPG. The differences observed with prosECCo75 are smaller due to the inclusion of electronic polarization effects. Another all-atom model, Slipids, also reveals a similar trend, except for POPC exhibiting higher line tension than POPS. This observation disagrees with available experimental data,^67,68^ which found that POPS lipids exhibit greater resistance to pore formation compared to POPC lipids. The observed discrepancy may be attributed to limitations in the Slipids force field, particularly its representation of lipid head groups and their interactions with ions, as demonstrated by comparison between MD simulations and NMR data.^63,69^ Therefore, these shortcomings can significantly impact the calculated line tension for lipid bilayers of different compositions.

Similarly, the polarizable version of coarse-grained Martini 2.2 model shows the lowest line tension for POPS, in disagreement with the experiments. Martini 2.2 and Martini 3 force fields also exhibit relatively poor performance, with POPG showing higher line tensions than POPC, which contradicts the experiments^53,65,66^ and mirrors the results obtained with the Full-Path method. Nevertheless, both Martini 2.2 and Martini 3 suggest that POPE has the highest line tension (in agreement with all-atom simulations), and, unlike Martini 2.2p, predict that POPS has a higher line tension than POPC. The latter observation suggests that the polarizable coarse-grained water model in Martini 2.2p has a major influence on line tension predictions.

To further assess the agreement between simulations and experiments, we directly compared the line tension values for POPC and POPC:POPG lipid mixtures with the experimental data reported in ref (53). As shown in Figure 6A, the all-atom force fields (including Slipids, which does not perform well in the case of POPC vs POPS) exhibit robust agreement with the experimental data. Meanwhile, the polarizable Martini 2.2p force field captures the experimental trend only qualitatively, whereas Martini 2.2 and Martini 3 fail to reproduce even the experimental trend.

We also checked the cross-agreement between the Full-Path and Rapid methods, Figure 6B. While the overall agreement between the two methods is excellent, the existing minor deviations are specific to the force field used. For instance, simulations employing the Martini 2.2 and Martini 2.2p force fields consistently yield slightly higher line tension predictions when using the Rapid method compared to the Full-Path method. In contrast, CHARMM36 simulations with the Rapid method tend to produce slightly lower line tension values. These discrepancies might be attributed to a small difference of ionic concentrations in two methods (0.15 m vs 0.15 M in the Rapid and Full-Path methods, respectively) and to the different geometry/size of pores (finite vs “infinite” radius). In addition, all-atom and coarse-grained force fields differ in their ability to capture membrane curvature, with even different versions of the coarse-grained Martini force field producing varying results.^70^

Next, we conducted a systematic analysis of the effects of ions on the line tension of lipid membranes using the Rapid method. Previous study by Lira et al.^53^ demonstrated that the addition of CaCl_2_ to giant unilamellar vesicles (GUVs) composed of an equimolar POPC:POPG mixture increases the line tension to levels exceeding that of pure POPC (see the left panel of Figure 7A). Our simulations, performed using the CHARMM36, prosECCo75, and Martini 2.2p force fields—all of which performing well for POPC:POPG mixtures, cf. Figure 6A—reliably reproduce this behavior, see Figure 7A. Although the absolute values of line tension visibly differ between the experimental and simulation data due to major differences in ionic and even lipid concentrations, the CHARMM36 force field accurately captures the observed experimental trend in line tension upon CaCl_2_ addition. Similarly, the prosECCo75 force field produces comparable results; however, the difference in line tension between pure POPC and POPC:POPG 1:1 mixture is insignificant. The Martini 2.2p force field also captures the overall behavior, although the presence of CaCl_2_ did not restore the line tension to levels equivalent to or higher than those of pure POPC.

Finally, to further compare our predictions with experimental data, we systematically investigated the effect of NaCl on the calculated line tensions of lipid membranes. Recent experimental works have shown that increasing the concentration of NaCl salt can substantially elevate the line tension of GUVs composed of egg phosholipid^71^ or DOPC:DOPG mixture.^72^ Here, we studied two scenarios: (i) the system contains 0.15 m NaCl, i.e., a concentration approximating physiological conditions, and (ii) no ions are present (or only sodium counterions when required). All results presented so far have been derived from simulations under the former condition. As shown in Figure 7B, the addition of NaCl significantly increases the line tension of membranes containing anionic lipids. Simulations using the all-atom CHARMM36 and prosECCo75 force fields indicate that this increase can range from 30 to 50%. The polarizable coarse-grained Martini 2.2p model shows a more moderate increase of up to 20%. Interestingly, the Martini 2.2, Martini 3, and the Slipids force field, which exhibit different limitations in accurately predicting the line tension and its changes, demonstrate smaller to no consistent effect on line tension upon the addition of NaCl. These observations suggest that the Slipids, Martini 2.2, and Martini 3 force fields may not be suitable for accurately predicting line tensions changes upon adding a physiological amount of salt, which also raises uncertainty about their accuracy in simulations involving varying ionic concentrations. In contrast, the CHARMM36 and prosECCo75 force fields, along with the Martini 2.2p model to some extent, appear more appropriate for this purpose, providing a closer agreement with experimental observations.

## Conclusions

Understanding pore formation in cellular membranes at the molecular level is a key to uncovering fundamental biological processes and facilitating various biomedical applications. This study integrates advanced computational techniques with MD simulations to obtain the free energy profiles of pore formation in lipid bilayers. We introduced two novel collective variables (CVs)—coined Full-Path and Rapid, respectively—which accurately describe the process of pore formation, stability, and closure, and can be used to evaluate the associated free energy changes. The Full-Path method tracks pore nucleation and expansion by focusing on the distribution of lipid tails within the pore region. This definition is based on our unbiased simulations, which suggest that lipid tails play an important role in the process of pore formation. We confirmed the robustness of our CV by demonstrating no hysteresis in the forward and backward free energy profiles. The Rapid method offers a more computationally efficient way to assess membrane line tension and calculate the free energy for larger pores. This method is based on the simulation biasing the box dimension corresponding to the pore rim. Both methods strongly align with reported experimental trends, particularly in predicting line tension variations due to lipid composition and ion concentration. Using our CVs, we assessed the performance of different force fields in predicting line tension. The all-atom force fields CHARMM36 and prosECCo75 consistently showed the best agreement with experimental data. Other force fields, such as all-atom Slipids and coarse-grained Martini 2.2, Martini 2.2 polarizable, and Martini 3, demonstrated variable performance depending on the lipids modeled. Both CVs were implemented using PLUMED, making them freely available and adaptable across different MD simulation engines. These CVs provide a robust alternative to existing approaches and can be readily applied to study pore formation mechanisms induced by external perturbations such as mechanical stresses or pore-forming agents.

## Data Availability

All the necessary files to reproduce our data, including topologies, force field parameters, and input configurations, are openly available on Zenodo at DOI: 10.5281/zenodo.13950778. The simulation protocols are thoroughly described in the manuscript and Supporting Information. The data were analyzed using GROMACS and PLUMED in-built tools, custom Python scripts, and Microsoft Excel. Plots andfigures were prepared using gnuplot, Python library Matplotlib, Microsoft Powerpoint, and GIMP software. Molecular structures were visualized using VMD. All software used is free of charge. CHARMM-GUI is available at https://www.charmm-gui.org/. GROMACS can be downloaded from https://www.gromacs.org/. PLUMED can be downloaded from https://www.plumed.org/. Python can be downloaded from https://www.python.org/. Microsoft Office software can be used online at https://www.office.com/. Gnuplot can be downloaded from http://www.gnuplot.info/. GIMP can be downloaded from https://www.gimp.org/. VMD can be downloaded from http://www.ks.uiuc.edu/Research/vmd/.

## Supporting Information Available

Supplementary Methods, Tables S1–S13, and Figures S1–S7, which include the line tension values calculated with the Rapid method, the backward free energy profile showing the absence of hysteresis with the Full-Path method, the correlation between the pore lifetime and the density of fatty acid carbons during spontaneous pore closure simulations, the distributions of CV values at the junction between membrane defect and membrane pore, a comparison of formed water channels depending on the radius of the cylinder in the Full-Path method, the values of line tension and quadratic coefficient from the Full-Path method for other lipids mixtures than POPC:POPG, and the relation between the quadratic coefficient and the line tension (PDF)
