# Supplementary material for: Free Energy of Membrane Pore Formation and Stability from Molecular Dynamics Simulations
Source: J Chem Inf Model. 2025 Jan 10;65(2):908–20. doi: 10.1021/acs.jcim.4c01960 (PMC11776052; doi:10.1021/acs.jcim.4c01960)
Supplement: Supplementary file 1 — ci4c01960_si_001.pdf [file ci4c01960_si_001.pdf]

Supporting Information: Free Energy of Membrane Pore Formation and Stability from Molecular Dynamics Simulations

Timothée Rivel,[†,§] Denys Biriukov,[†,‡,§] Ivo Kabelka,[†] and Robert Vácha[*,†,¶,‡]

†*Central European Institute of Technology, Masaryk University, Kamenice 5, CZ-62500 Brno, Czech Republic*

‡*National Centre for Biomolecular Research, Faculty of Science, Masaryk University, Kamenice 5, CZ-62500 Brno, Czech Republic*

¶*Department of Condensed Matter Physics, Faculty of Science, Masaryk University, Kotlářská 267/2, CZ-61137 Brno, Czech Republic*

§*These authors contributed equally to this work and are allowed to change the publication order to list them as first in their CVs*

E-mail: robert.vacha@muni.cz

Supplementary Methods

Setting Up and Equilibration of Lipid stripes

To prepare a lipid stripe suitable for simulations with the *Rapid* method, each bilayer was first equilibrated following the protocol recommended by CHARMM-GUI.[S1–S3] The equilibrated bilayer, Figure S1A, was then placed in a larger simulation box by extending one of the membrane's lateral dimensions. The system was resolvated with water, and ions were added when necessary.

Subsequently, the system underwent energy minimization, followed by a series of equilibration runs. The first equilibration was a short 100 ps *NVT* run with a 1 fs time step (5 fs for Martini). During this run, position restraints (with a force constant $k_{pr} = 1000\ \mathrm{kJ \cdot mol^{-1} \cdot nm^{-2}}$) were applied in all directions to the phosphorus atoms (or Martini beads, $k_{pr} = 100\ \mathrm{kJ \cdot mol^{-1} \cdot nm^{-2}}$) and, in some cases, to the terminal carbons of the lipid tails. Dihedral restraints ($k_{dihr} = 1000\ \mathrm{kJ \cdot mol^{-1} \cdot rad^{-2}}$), as suggested by CHARMM-GUI, were also applied in the case of all-atom simulations. Next, a 250 ps *NPT* run with a 1 fs time step (5 fs for Martini) and the same restraints (all-atom: $k_{pr} = 400\ \mathrm{kJ \cdot mol^{-1} \cdot nm^{-2}}$, $k_{dihr} = 200\ \mathrm{kJ \cdot mol^{-1} \cdot rad^{-2}}$; Martini: $k_{pr} = 50\ \mathrm{kJ \cdot mol^{-1} \cdot nm^{-2}}$) was conducted using an anisotropic barostat. This barostat acted independently only along the membrane's lateral dimensions, *i.e.*, along the extended dimension and parallel to the pore rim. The resulting structure is shown in Figure S1B.

Following this, the position and dihedral restraints were replaced with flat-bottom restraints designed to prevent the lipid stripe from rotating, as described in the main text. A subsequent 500 ps *NVT* simulation with a 1 fs time step (20 fs for Martini) was performed, leading to the formation or initial development of the lipid stripe, Figure S1C. To generate starting configurations for umbrella sampling (US), the lipid stripe was compressed along the pore-rim dimension during an *NPT* simulation lasting up to 500 ps with a 1 fs time step (20 fs for Martini). In this simulation, the pressure along the pore rim was set to 500 bar

(75 bar for Martini). All equilibration simulations were carried out using the Berendsen thermostat[S4] with a coupling time of 1 ps (v-rescale thermostat[S5] with a coupling time of 1 ps for Martini) and the Berendsen barostat[S4] with a coupling time of 5 ps.

From the "pressing" simulation, configurations of lipid stripes with pore rim sizes ranging from 6 nm to 6.6 nm were extracted. Each US window was then equilibrated for 1 ns with 2 fs time step (20 fs for Martini) in the *NVT* ensemble using the Nosé–Hoover thermostat[S6,S7] with a coupling time of 1 ps (except for Slipids, where v-rescale thermostat[S5] with a 0.5 ps coupling time was used). Figure S1D shows an example of an equilibrated lipid stripe. After equilibration, the production runs were conducted as detailed in the main text. All other details of the simulation protocols for equilibration simulations followed the force field recommendations and were consistent with those used in the production runs.

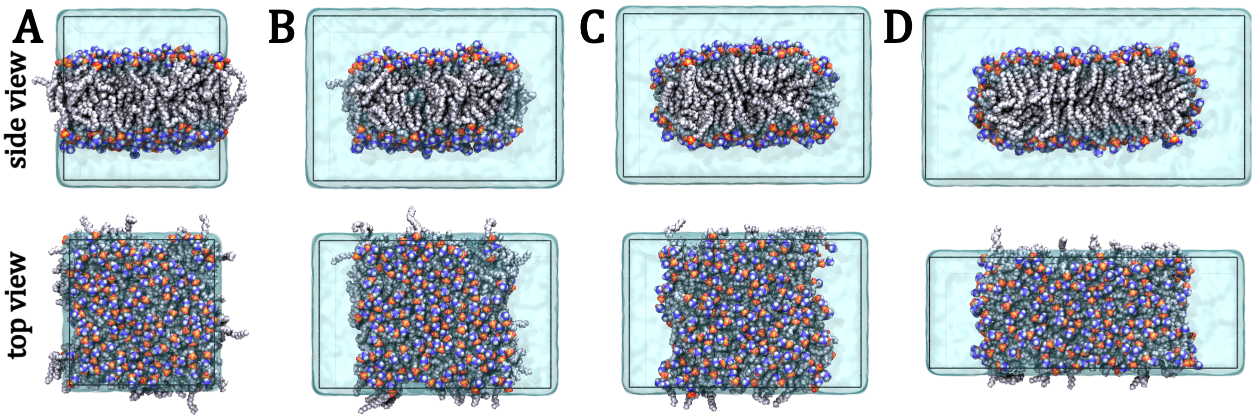

Figure S1: The illustration depicting the process of preparing and equilibrating a lipid stripe: A) A lipid bilayer is initially constructed and equilibrated. B) The bilayer is then placed in a larger simulation box and equilibrated with restrained lipids. C) The positional restraints are removed, allowing the lipid head groups to reorient covering the lipid tails from water and leading to the formation or initial development of a lipid stripe. D) The bilayer is then compressed to generate structures with various size of a membrane rim.

Supplementary Data

Table S1: Summary of the unbiased simulations of spontaneous pore closure events. The table lists the number of simulation replicas analyzed for each system. A system was included in the analysis if a pore closure event occurred during the simulation, without transitioning to the gel phase. Simulations were conducted for 300–1000 ns or until the membrane showed no evidence of an open pore.

	DMPC	DPPC	POPC	DOPC
CHARMM36	1	3	5	5
Slipids	10	10	5	5
Lipid14	0	0	5	5
Martini 2.2	20	20	20	20
Berger	10	0	4	5

Table S2: Summary of the simulation parameters utilized during the production runs with all-atom force fields.

	CHARMM36[S8,S9] & prosECCo75[S10]	Slipids[S11–S14*]	Lipid14[S15]	Berger[S16]
Electrostatics	PME[S17,S18]	PME[S17,S18]	PME[S17,S18]	PME[S17,S18]
Coulomb cutoff [nm]	1.2	1.4	1.0	1.2
LJ cutoff [nm]	1.2	1.4	1.0	1.2
LJ modifier	Force switch at 1.0–1.2 nm	Potential shift	Potential shift	Potential shift
Dispersion correction	–	Energy & pressure[S19]	Energy & pressure[S19]	Energy & pressure[S19]
Thermostat	Nosé–Hoover[S6,S7]	V-rescale[S5]	Nosé–Hoover[S6,S7]	Nosé–Hoover[S6,S7]
Coupling constant (T) [ps]	1	0.5	0.5	0.5
Barostat	Parrinello–Rahman[S20]	Berendsen[S4]	Berendsen[S4]	Parrinello–Rahman[S20]
Coupling constant (P) [ps]	5	10	1	2
Compressibility (P) [1/bar]	$4.5 \cdot 10^{-5}$	$4.5 \cdot 10^{-5}$	$4.5 \cdot 10^{-5}$	$4.5 \cdot 10^{-5}$
Constraints	Bonds with H	All bonds	Bonds with H	All bonds
Constraint algorithm	P-LINCS[S21,S22]	P-LINCS[S21,S22]	P-LINCS[S21,S22]	P-LINCS[S21,S22]

*) For spontaneous pore closure simulations: Coulomb and LJ cutoffs = 1.2 nm; thermostat – Nosé–Hoover;[S6,S7] barostat – Parrinello–Rahman.[S20]

Table S3: Summary of the simulation parameters utilized during the production runs with the Martini family of coarse-grained force fields.

	Martini 2.2[S23] & Martini 3[S24]	Martini 2.2p[S25]
Electrostatics	Reaction field[S26]	Reaction field[S26]
Relative dielectric constant	15	2.5
Coulomb cutoff [nm]	1.1	1.1
LJ cutoff [nm]	1.1	1.1
LJ modifier	Potential shift	Potential shift
Dispersion correction	–	–
Thermostat	V-rescale[S5]	V-rescale[S5]
Coupling constant (T) [ps]	1.0	1.0
Barostat	Parrinello–Rahman[S20]	Parrinello–Rahman[S20]
Coupling constant (P) [ps]	12.0	12.0
Compressibility (P) [1/bar]	$3 \cdot 10^{-4}$	$3 \cdot 10^{-4}$
Constraints	–	–

Table S4: Summary of the tested lipid composition and force field combinations using the *Full-Path* method. Each combination was modeled in a physiological 0.15 M NaCl concentration, with additional sodium cations added as needed to neutralize a net charge of negatively charged lipids.

Composition	Name	CHARMM36	Martini 2.2	Martini 3	Martini 2.2p
POPC	PC	✓	✓	✓	✓
POPC:POPG 3:1	PC-PG-3-1				✓
POPC:POPG 1:1	PC-PG-1-1				✓
POPC:POPG 1:3	PC-PG-1-3				✓
POPG	PG	✓	✓	✓	✓
POPE	PE		✓		✓
POPE:POPG 3:1	PE-PG-3-1		✓		✓
POPE:POPG 2:1	PE-PG-2-1				✓
POPE:POPG 1:1	PE-PG-1-1				✓
POPS	PS				✓
POPC:POPS 3:1	PC-PS-3-1				✓
POPC:POPS 1:1	PC-PS-1-1				✓

Table S5: Summary of the lipid composition and force field combinations tested for line tension predictions using the *Rapid* method. Each combination was modeled under two conditions: in pure water or in a physiological 0.15 m NaCl concentration, with additional sodium cations added as needed to neutralize a net charge of negatively charged lipids. Additionally, each combination marked with a star was also modeled in 0.15 m $CaCl_2$ concentration.

Composition	Name	CHARMM36	prosECCo75	Slipids	Martini 2.2	Martini 3	Martini 2.2p
POPC	PC	✓	✓	✓	✓	✓	✓
POPC:POPG 3:1	PC-PG-3-1	✓	✓	✓	✓	✓	✓
POPC:POPG 1:1	PC-PG-1-1	✓*	✓*	✓	✓	✓	✓*
POPC:POPG 1:3	PC-PG-1-3	✓	✓		✓	✓	✓
POPG	PG	✓	✓	✓	✓	✓	✓
POPE	PE	✓	✓	✓	✓	✓	✓
POPE:POPG 3:1	PE-PG-3-1	✓					✓
POPE:POPG 1:1	PE-PG-3-1	✓					✓
POPE:POPG 1:3	PE-PG-3-1	✓					✓
POPS	PS	✓	✓	✓	✓	✓	✓

Table S6: Simulated all-atom systems for line tension predictions using the *Rapid* method.

Force Field	System Name	№$_{POPC}$	№$_{POPG}$	№$_{POPE}$	№$_{POPS}$	№$_{water/beads}$	№$_{Na^+}$	№$_{Ca^{2+}}$	№$_{Cl^-}$
CHARMM36 / prosECCo75	PC	200	–	–	–	20000	–	–	–
	PC-PG-3-1	150	50	–	–	20000	50	–	–
	PC-PG-1-1	100	100	–	–	20000	100	–	–
	PC-PG-1-3	50	150	–	–	20000	150	–	–
	PG	–	200	–	–	20000	200	–	–
CHARMM36 / prosECCo75	PC-NaCl	200	–	–	–	20000	54	–	54
	PC-PG-3-1-NaCl	150	50	–	–	20000	104	–	54
	PC-PG-1-1-NaCl	100	100	–	–	20000	154	–	54
	PC-PG-1-1-$CaCl_2$	100	100	–	–	20000	100	54	108
	PC-PG-1-3-NaCl	50	150	–	–	20000	204	–	54
	PG-NaCl	–	200	–	–	20000	254	–	54
CHARMM36	PE	–	–	200	–	20000	–	–	–
	PE-PG-3-1	–	50	150	–	20000	50	–	–
	PE-PG-1-1	–	100	100	–	20000	100	–	–
	PE-PG-1-3	–	150	50	–	20000	150	–	–
	PS	–	–	–	200	20000	200	–	–
CHARMM36	PE-NaCl	–	–	200	–	20000	54	–	54
	PE-PG-3-1-NaCl	–	50	150	–	20000	104	–	54
	PE-PG-1-1-NaCl	–	100	100	–	20000	154	–	54
	PE-PG-1-3-NaCl	–	150	50	–	20000	204	–	54
	PS-NaCl	–	–	–	200	20000	254	–	54
prosECCo75	PE	–	–	200	–	20000	–	–	–
	PS	–	–	–	200	20000	200	–	–
	PE-NaCl	–	–	200	–	20000	54	–	54
	PS-NaCl	–	–	–	200	20000	254	–	54
Slipids	PC	200	–	–	–	20000	–	–	–
	PC-PG-3-1	150	50	–	–	20000	50	–	–
	PC-PG-1-1	100	100	–	–	20000	100	–	–
	PG	–	200	–	–	20000	200	–	–
	PE	–	–	200	–	20000	–	–	–
	PS	–	–	–	200	20000	200	–	–
Slipids	PC-NaCl	200	–	–	–	20000	54	–	54
	PC-PG-3-1-NaCl	150	50	–	–	20000	104	–	54
	PC-PG-1-1-NaCl	100	100	–	–	20000	154	–	54
	PG-NaCl	–	200	–	–	20000	254	–	54
	PE-NaCl	–	–	200	–	20000	54	–	54
	PS-NaCl	–	–	–	200	20000	254	–	54

Table S7: Simulated coarse-grained systems for line tension predictions using the *Rapid* method.

Force Field	System Name	№$_{POPC}$	№$_{POPG}$	№$_{POPE}$	№$_{POPS}$	№$_{water/beads}$	№$_{Na^+}$	№$_{Ca^{2+}}$	№$_{Cl^-}$
Martini 2.2 / Martini 3 / Martini 2.2p	PC	200	–	–	–	5000	–	–	–
	PC-PG-3-1	150	50	–	–	5000	50	–	–
	PC-PG-1-1	100	100	–	–	5000	100	–	–
	PC-PG-1-3	50	150	–	–	5000	150	–	–
	PG	–	200	–	–	5000	200	–	–
Martini 2.2 / Martini 3 / Martini 2.2p	PC-NaCl	200	–	–	–	5000	54	–	54
	PC-PG-3-1-NaCl	150	50	–	–	5000	104	–	54
	PC-PG-1-1-NaCl	100	100	–	–	5000	154	–	54
	PC-PG-1-3-NaCl	50	150	–	–	5000	204	–	54
	PG-NaCl	–	200	–	–	5000	254	–	54
Martini 2.2 / Martini 3 / Martini 2.2p	PE	–	200	–	–	5000	–	–	–
	PS	–	–	–	200	5000	200	–	–
	PE-NaCl	–	200	–	–	5000	54	–	54
	PS-NaCl	–	–	–	200	5000	254	–	54
Martini 2.2p	PE-PG-3-1	–	50	150	–	5000	50	–	–
	PE-PG-1-1	–	100	100	–	5000	100	–	–
	PE-PG-1-3	–	150	50	–	5000	150	–	–
Martini 2.2p	PE-PG-3-1-NaCl	–	50	150	–	5000	104	–	54
	PE-PG-1-1-NaCl	–	100	100	–	5000	154	–	54
	PE-PG-1-1-$CaCl_2$	–	100	100	–	5000	100	54	108
	PE-PG-1-3-NaCl	–	150	50	–	5000	204	–	54

*) In case of Martini 2.2, 500 water beads were replaced by antifreeze type water beads.

Table S8: Line tension predictions from CHARMM36 simulations using the *Rapid* method. Error represents half the difference between estimates from the first and second halves of production simulations.

Force Field	System Name	Line Tension [pN]
CHARMM36	PC	32.5 ± 0.5
	PC-PG-3-1	29.4 ± 0.5
	PC-PG-1-1	26.1 ± 0.7
	PC-PG-1-3	19.6 ± 0.9
	PG	14.0 ± 2.6
CHARMM36	PC-NaCl	34.1 ± 0.9
	PC-PG-3-1-NaCl	35.4 ± 0.8
	PC-PG-1-1-NaCl	29.2 ± 2.3
	PC-PG-1-1-$CaCl_2$	35.5 ± 0.5
	PC-PG-1-3-NaCl	27.8 ± 2.0
	PG-NaCl	21.4 ± 0.4
CHARMM36	PE	59.4 ± 1.6
	PE-PG-3-1	46.3 ± 0.8
	PE-PG-1-1	34.1 ± 2.8
	PE-PG-1-3	23.6 ± 0.6
	PS	32.4 ± 2.6
CHARMM36	PE-NaCl	59.2 ± 0.5
	PE-PG-3-1-NaCl	52.0 ± 0.4
	PE-PG-1-1-NaCl	39.7 ± 1.5
	PE-PG-1-3-NaCl	28.5 ± 0.1
	PS-NaCl	44.5 ± 3.2

Table S9: Line tension predictions from prosECCo75 simulations using the *Rapid* method. Error represents half the difference between estimates from the first and second halves of production simulations.

Force Field	System Name	Line Tension [pN]
prosECCo75	PC	43.9 ± 0.7
	PC-PG-3-1	38.6 ± 1.0
	PC-PG-1-1	34.8 ± 0.9
	PC-PG-1-3	30.7 ± 1.6
	PG	25.8 ± 1.9
prosECCo75	PC-NaCl	41.1 ± 1.7
	PC-PG-3-1-NaCl	40.7 ± 0.2
	PC-PG-1-1-NaCl	41.1 ± 0.6
	PC-PG-1-1-$CaCl_2$	43.9 ± 0.5
	PC-PG-1-3-NaCl	37.5 ± 1.4
	PG-NaCl	32.8 ± 0.3
prosECCo75	PE	52.6 ± 1.9
	PS	36.7 ± 0.7
	PE-NaCl	54.6 ± 0.4
	PS-NaCl	48.5 ± 0.6

Table S10: Line tension predictions from Slipids simulations using the *Rapid* method. Error represents half the difference between estimates from the first and second halves of production simulations.

Force Field	System Name	Line Tension [pN]
Slipids	PC	42.4 ± 1.3
	PC-PG-3-1	36.4 ± 2.5
	PC-PG-1-1	33.1 ± 0.4
	PG	28.2 ± 1.3
	PE	59.8 ± 0.0
	PS	28.5 ± 0.8
Slipids	PC-NaCl	41.0 ± 1.4
	PC-PG-3-1-NaCl	38.2 ± 0.2
	PC-PG-1-1-NaCl	36.8 ± 0.7
	PG-NaCl	29.4 ± 1.4
	PE-NaCl	60.0 ± 1.3
	PS-NaCl	32.6 ± 2.2

Table S11: Line tension predictions from Martini 2.2 simulations using the *Rapid* method. Error represents half the difference between estimates from the first and second halves of production simulations.

Force Field	System Name	Line Tension [pN]
Martini 2.2	PC	57.7 ± 0.5
	PC-PG-3-1	61.6 ± 0.4
	PC-PG-1-1	65.0 ± 0.8
	PC-PG-1-3	65.8 ± 0.1
	PG	64.5 ± 0.1
Martini 2.2	PC-NaCl	57.3 ± 0.0
	PC-PG-3-1-NaCl	60.1 ± 0.9
	PC-PG-1-1-NaCl	63.4 ± 0.6
	PC-PG-1-3-NaCl	64.4 ± 0.4
	PG-NaCl	65.1 ± 0.3
Martini 2.2	PE	77.2 ± 1.0
	PS	60.5 ± 0.2
	PE-NaCl	76.2 ± 1.0
	PS-NaCl	61.9 ± 1.0

Table S12: Line tension predictions from Martini 3 simulations using the *Rapid* method. Error represents half the difference between estimates from the first and second halves of production simulations.

Force Field	System Name	Line Tension [pN]
Martini 3	PC	47.9 ± 0.1
	PC-PG-3-1	50.3 ± 0.2
	PC-PG-1-1	52.8 ± 1.0
	PC-PG-1-3	51.9 ± 0.0
	PG	54.3 ± 1.3
Martini 3	PC-NaCl	49.4 ± 0.2
	PC-PG-3-1-NaCl	53.4 ± 0.1
	PC-PG-1-1-NaCl	54.1 ± 2.0
	PC-PG-1-3-NaCl	54.3 ± 0.5
	PG-NaCl	53.7 ± 1.0
Martini 3	PE	61.6 ± 1.3
	PS	52.7 ± 0.2
	PE-NaCl	61.1 ± 0.3
	PS-NaCl	55.4 ± 0.5

Table S13: Line tension predictions from Martini 2.2p simulations using the *Rapid* method. Error represents half the difference between estimates from the first and second halves of production simulations.

Force Field	System Name	Line Tension [pN]
Martini 2.2p	PC	46.8 ± 0.7
	PC-PG-3-1	44.6 ± 0.1
	PC-PG-1-1	40.8 ± 1.2
	PC-PG-1-3	36.8 ± 0.2
	PG	32.6 ± 0.2
Martini 2.2p	PC-NaCl	49.2 ± 0.6
	PC-PG-3-1-NaCl	48.1 ± 0.8
	PC-PG-1-1-NaCl	46.4 ± 0.2
	PC-PG-1-1-$CaCl_2$	48.6 ± 0.1
	PC-PG-1-3-NaCl	42.0 ± 0.6
	PG-NaCl	38.8 ± 0.6
Martini 2.2p	PE	53.6 ± 1.3
	PS	28.4 ± 0.4
	PE-NaCl	55.7 ± 0.6
	PS-NaCl	34.3 ± 0.4
Martini 2.2p	PE-PG-3-1	49.9 ± 0.0
	PE-PG-1-1	44.7 ± 1.3
	PE-PG-1-3	37.9 ± 0.8
Martini 2.2p	PE-PG-3-1-NaCl	54.1 ± 0.1
	PE-PG-1-1-NaCl	49.8 ± 0.5
	PE-PG-1-3-NaCl	43.3 ± 0.7

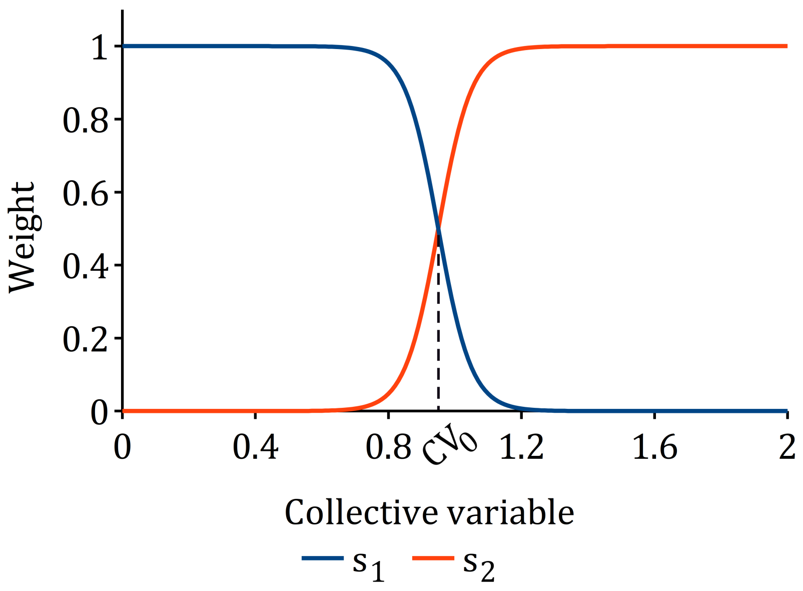

Figure S2: The switching functions s_1 and s_2 that are weighing CV_{cyl} and CV_{radius}, respectively.

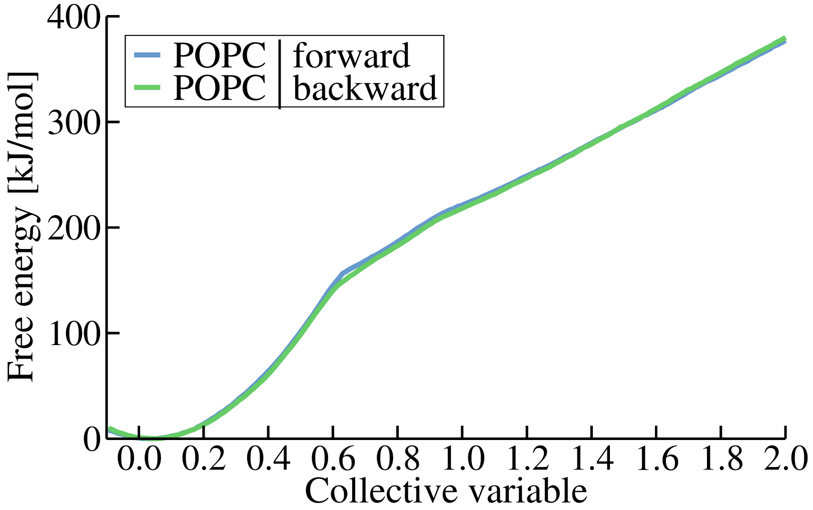

Figure S3: The free energy profiles obtained with the *Full-Path* method for the POPC membrane simulated with the Martini 2.2p force field. A comparison between the forward and backward pulling simulations used to generate the umbrella sampling windows reveals no noticeable differences between the two profiles.

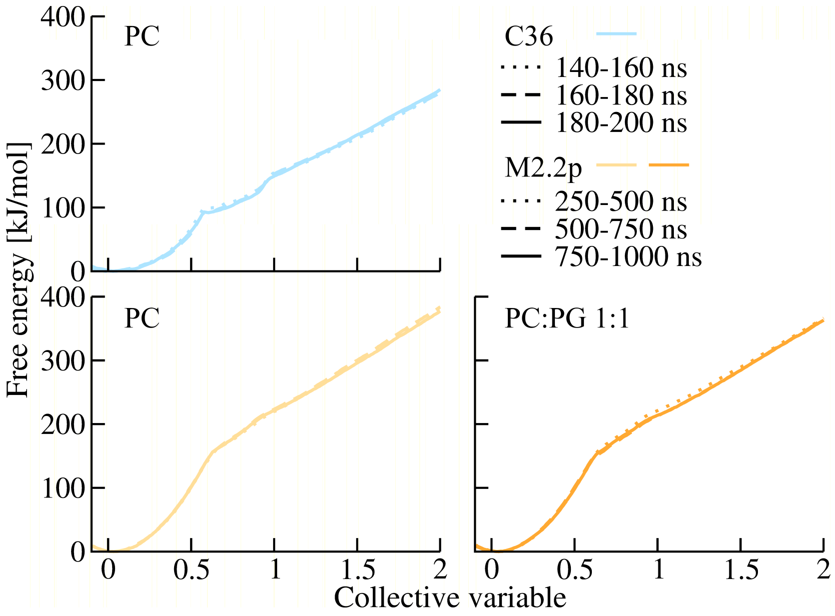

Figure S4: The free energy profiles obtained with the *Full-Path* method for POPC and POPC:POPG 1:1 membranes simulated with CHARMM36 and Martini 2.2p force fields. A comparison between the free energy profiles using different 20 ns parts of the trajectory shows the good convergence of our free energy calculations using both all-atom and coarse-grained force fields.

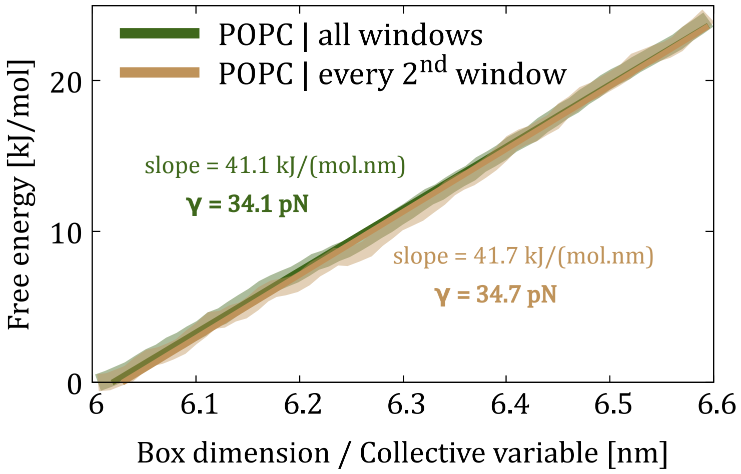

Figure S5: Free energy profiles obtained with the *Rapid* method using either all 21 umbrella sampling windows or only every second window. The comparison is given for a POPC lipid stripe in a 0.15 m NaCl solution, simulated with the CHARMM36 force field.

Carbon	R-Squared	Pearson	Spearman	Kendall
C_{-1}	0.82	0.91	0.95	0.82
C_{-2}	0.62	0.79	0.84	0.64
C_{-3}	0.50	0.71	0.74	0.59

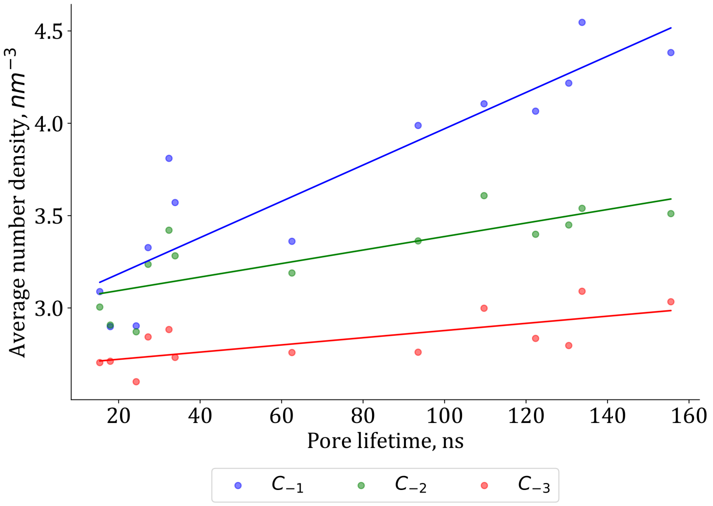

Figure S6: The plot shows the positive correlation between the pore lifetime and the density of the last carbon atoms of the lipid tails. C_{-1} refers to the carbon atom at the very end of the lipid tail, C_{-2} refers to the second-to-last carbon, and C_{-3} refers to the third-to-last carbon. The table reports the respective values of the R-squared correlation coefficient, the Pearson correlation coefficient, the Spearman Rank correlation coefficient, and the Kendall's tau correlation coefficient.

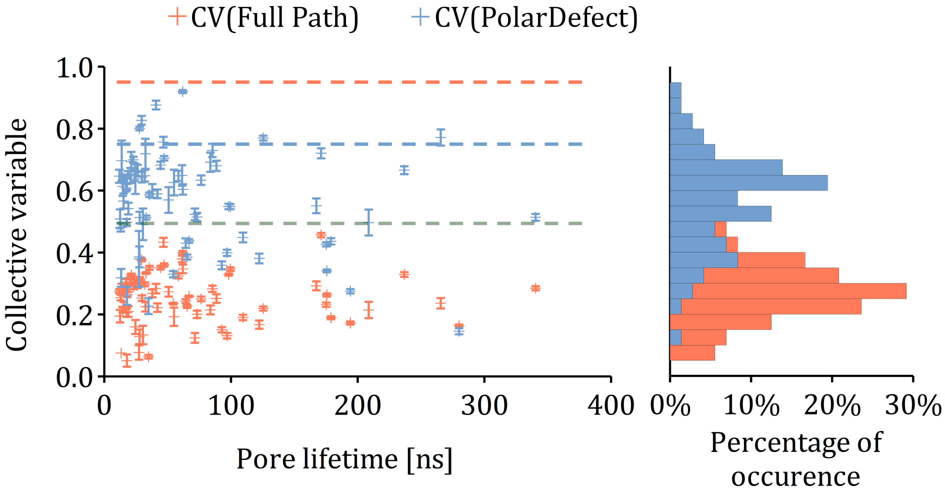

Figure S7: Left panel: Values of two CVs measured during all-atom MD simulations of spontaneous pore closure. CV(Full Path) refers to the *Full-Path* CV for pore formation introduced in this work, while CV(PolarDefect) refers to our PLUMED implementation of the pore nucleation CV presented in ref S27. Each point represents an average of 11 data points (five before and five after the transition) centered on the frame where the pore lifetime was calculated (using the state of the pore $s(t)$ observable). The data spans a 200 ps window around the pore closure event, with error bars indicating the standard error. The horizontal red and blue dashed lines show the CV values at which the junction to the pore expansion part of the CV occurs—either for our *Full-Path* CV or for the revised joint CV presented in ref S28. The horizontal green dashed line is plotted at 0.5 as a visual aid, indicating that all pore closure events for the *Full-Path* CV occur below this value. It should be noted that for values below ~0.7, $CV \approx CV_{cyl}$, as shown in Figure S2. Right panel: Distribution (in percentages) of the CV values observed during spontaneous pore closure simulations. The data are binned in 0.05 intervals.

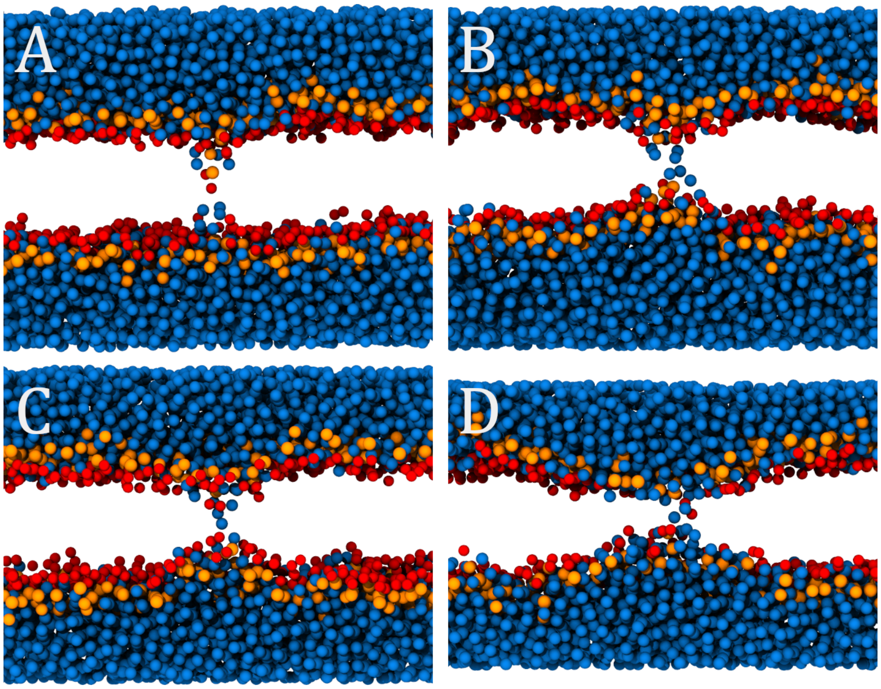

Figure S8: Comparison of water channels formed by the depletion of lipid tails inside the cylinders with radii of A) 0.5 nm, B) 0.75 nm, C) 1.5 nm, and D) 3 nm. Cross-sectional views of the membrane pores are shown. Water beads are shown in blue, while lipid heads and carbonyl groups are shown in orange and red, respectively. A) A water channel is not formed even after the displacement of all lipid tail atoms. B & C) The water channels resemble those formed in simulations of spontaneous pore closure. D) Significant membrane thinning in the vicinity of the initial water channel.

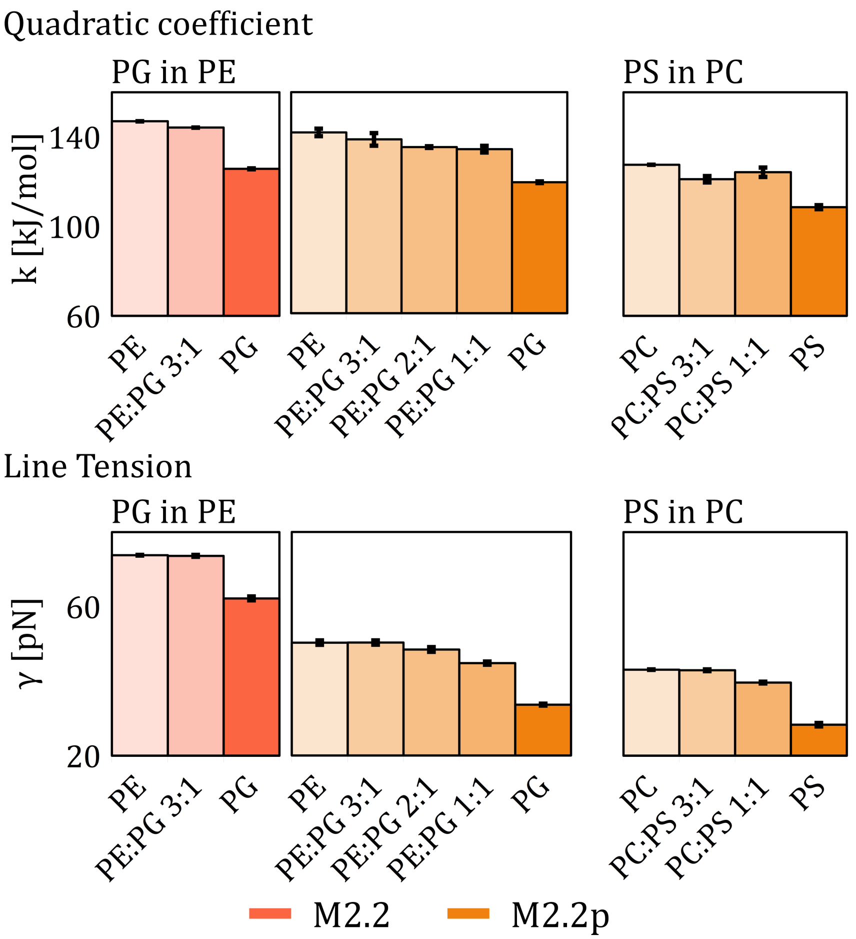

Figure S9: Line tension and quadratic coefficient calculated using the *Full-Path* method for pure POPE, POPC, and POPS, as well as POPE:POPG and POPC:POPS mixtures.

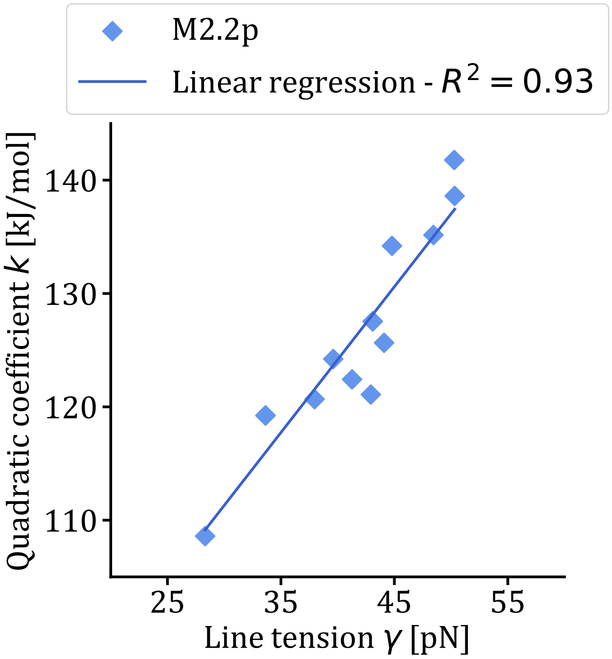

Figure S10: The relation of the quadratic coefficient k to the line tension γ. The data are calculated using the *Full-Path* method for pure POPC, POPE, POPG, POPS, as well as POPC:POPG, POPE:POPG, and POPC:POPS mixtures, all simulated with the Martini 2.2p force field.

References

(S1) Jo, S.; Lim, J. B.; Klauda, J. B.; Im, W. CHARMM-GUI membrane builder for mixed bilayers and its application to yeast membranes. *Biophys. J.* **2009**, *97*, 50–58.

(S2) Wu, E. L.; Cheng, X.; Jo, S.; Rui, H.; Song, K. C.; Dávila-Contreras, E. M.; Qi, Y.; Lee, J.; Monje-Galvan, V.; Venable, R. M.; Klauda, J. B.; Im, W. CHARMM-GUI membrane builder toward realistic biological membrane simulations. **2014**, *35*, 1997–2004.

(S3) Lee, J.; Cheng, X.; Swails, J. M.; Yeom, M. S.; Eastman, P. K.; Lemkul, J. A.; Wei, S.; Buckner, J.; Jeong, J. C.; Qi, Y.; Jo, S.; Pande, V. S.; Case, D. A.; Brooks, C. L.; MacKerell, A. D.; Klauda, J. B.; Im, W. CHARMM-GUI Input Generator for NAMD, GROMACS, AMBER, OpenMM, and CHARMM/OpenMM Simulations Using the CHARMM36 Additive Force Field. *J. Chem. Theory Comput.* **2016**, *12*, 405–413.

(S4) Berendsen, H. J.; Postma, J. P.; Van Gunsteren, W. F.; Dinola, A.; Haak, J. R. Molecular dynamics with coupling to an external bath. *J. Chem. Phys.* **1984**, *81*, 3684–3690.

(S5) Bussi, G.; Donadio, D.; Parrinello, M. Canonical sampling through velocity rescaling. *J. Chem. Phys.* **2007**, *126*, 014101.

(S6) Nosé, S. A molecular dynamics method for simulations in the canonical ensemble. *Mol. Phys.* **1984**, *52*, 255–268.

(S7) Hoover, W. G. Canonical dynamics: Equilibrium phase-space distributions. *Phys. Rev. A* **1985**, *31*, 1695–1697.

(S8) Klauda, J. B.; Venable, R. M.; Freites, J. A.; O'Connor, J. W.; Tobias, D. J.; Mondragon-Ramirez, C.; Vorobyov, I.; MacKerell, A. D.; Pastor, R. W. Update of

the CHARMM All-Atom Additive Force Field for Lipids: Validation on Six Lipid Types. *J. Phys. Chem. B* **2010**, *114*, 7830–7843.

(S9) Venable, R. M.; Luo, Y.; Gawrisch, K.; Roux, B.; Pastor, R. W. Simulations of anionic lipid membranes: Development of interaction-specific ion parameters and validation using NMR data. *J. Phys. Chem. B* **2013**, *117*, 10183–10192.

(S10) Nencini, R.; Tempra, C.; Biriukov, D.; Riopedre-Fernandez, M.; Chamorro, V. C.; Polák, J.; Mason, P. E.; Ondo, D.; Heyda, J.; Ollila, O. H. S.; Jungwirth, P.; Javanainen, M.; Martinez-Seara, H. Effective Inclusion of Electronic Polarization Improves the Description of Electrostatic Interactions: The prosECCo75 Biomolecular Force Field. *J. Chem. Theory Comput.* **2024**, *20*, 7546–7559.

(S11) Jämbeck, J. P.; Lyubartsev, A. P. An extension and further validation of an all-atomistic force field for biological membranes. *J. Chem. Theory Comput.* **2012**, *8*, 2938–2948.

(S12) Jämbeck, J. P.; Lyubartsev, A. P. Derivation and systematic validation of a refined all-atom force field for phosphatidylcholine lipids. *J. Phys. Chem. B* **2012**, *116*, 3164–3179.

(S13) Jämbeck, J. P.; Lyubartsev, A. P. Another piece of the membrane puzzle: Extending slipids further. *J. Chem. Theory Comput.* **2013**, *9*, 774–784.

(S14) Grote, F.; Lyubartsev, A. P. Optimization of Slipids Force Field Parameters Describing Headgroups of Phospholipids. *J. Phys. Chem. B* **2020**, *124*, 8784–8793.

(S15) Dickson, C. J.; Madej, B. D.; Skjevik, Å. A.; Betz, R. M.; Teigen, K.; Gould, I. R.; Walker, R. C. Lipid14: The amber lipid force field. *J. Chem. Theory Comput.* **2014**, *10*, 865–879.

(S16) Berger, O.; Edholm, O.; Jähnig, F. Molecular dynamics simulations of a fluid bilayer of dipalmitoylphosphatidylcholine at full hydration, constant pressure, and constant temperature. *Biophys. J.* **1997**, *72*, 2002–2013.

(S17) Darden, T.; York, D.; Pedersen, L. Particle mesh Ewald: An $N \cdot \log(N)$ method for Ewald sums in large systems. *J. Chem. Phys.* **1993**, *98*, 10089–10092.

(S18) Essmann, U.; Perera, L.; Berkowitz, M. L.; Darden, T.; Lee, H.; Pedersen, L. G. A smooth particle mesh Ewald method. *J. Chem. Phys.* **1995**, *103*, 8577–8593.

(S19) Shirts, M. R.; Mobley, D. L.; Chodera, J. D.; Pande, V. S. Accurate and efficient corrections for missing dispersion interactions in molecular simulations. *J. Phys. Chem. B* **2007**, *111*, 13052–13063.

(S20) Parrinello, M.; Rahman, A. Polymorphic transitions in single crystals: A new molecular dynamics method. *J. Appl. Phys.* **1981**, *52*, 7182–7190.

(S21) Hess, B.; Bekker, H.; Berendsen, H. J.; Fraaije, J. G. LINCS: A Linear Constraint Solver for molecular simulations. *J. Comput. Chem.* **1997**, *18*, 1463–1472.

(S22) Hess, B. P-LINCS: A parallel linear constraint solver for molecular simulation. *J. Chem. Theory Comput.* **2008**, *4*, 116–122.

(S23) Marrink, S. J.; Risselada, H. J.; Yefimov, S.; Tieleman, D. P.; De Vries, A. H. The MARTINI force field: Coarse grained model for biomolecular simulations. *J. Phys. Chem. B* **2007**, *111*, 7812–7824.

(S24) Souza, P. C.; Alessandri, R.; Barnoud, J.; Thallmair, S.; Faustino, I.; Grünewald, F.; Patmanidis, I.; Abdizadeh, H.; Bruininks, B. M.; Wassenaar, T. A.; Kroon, P. C.; Melcr, J.; Nieto, V.; Corradi, V.; Khan, H. M.; Domański, J.; Javanainen, M.; Martinez-Seara, H.; Reuter, N.; Best, R. B.; Vattulainen, I.; Monticelli, L.; Peri-

ole, X.; Tieleman, D. P.; de Vries, A. H.; Marrink, S. J. Martini 3: a general purpose force field for coarse-grained molecular dynamics. *Nat. Methods* **2021**, *18*, 382–388.

(S25) Yesylevskyy, S. O.; Schäfer, L. V.; Sengupta, D.; Marrink, S. J. Polarizable water model for the coarse-grained MARTINI force field. *PLoS Comput. Biol.* **2010**, *6*, 1–17.

(S26) Tironi, I. G.; Sperb, R.; Smith, P. E.; Van Gunsteren, W. F. A generalized reaction field method for molecular dynamics simulations. *J. Chem. Phys.* **1995**, *102*, 5451–5459.

(S27) Hub, J. S.; Awasthi, N. Probing a Continuous Polar Defect: A Reaction Coordinate for Pore Formation in Lipid Membranes. *J. Chem. Theory Comput.* **2017**, *13*, 2352–2366.

(S28) Hub, J. S. Joint Reaction Coordinate for Computing the Free-Energy Landscape of Pore Nucleation and Pore Expansion in Lipid Membranes. *J. Chem. Theory Comput.* **2021**, *17*, 1229–1239.
